# Supplementary material for: Molecular detection and antibiotic resistance of diarrheagenic Escherichia coli from street food and water in mukuru slums, Nairobi County
Source: PLoS One. 2026 Jan 28;21(1):e0340081. doi: 10.1371/journal.pone.0340081 (PMC12851472; doi:10.1371/journal.pone.0340081)
Supplement: S1 Fig — (ZIP) [file pone.0340081.s001.zip › Updated Supporting Information/S1.Fig The Ingredients and Description of the Street Foods (1).docx]

**Manuscript Supporting Information**

**S1 Fig : The Ingredients and Description of the Street Foods collected and analyzed from Mukuru Informal Settlement Nairobi Kenya.**

| **Food Item** | **Ingredients** | **Description** |
| --- | --- | --- |
| *Mandazi* | Wheat dough, cooking oil, baking  powder, salt, Sugar | It is a deep fried piece of wheat dough commonly with a triangle or rectangular shape that is deep fried until golden brown. |
| *Fries* | Rectangular tube shaped piece of  Potato. | Sliced extremely thin and then fired until they become crisp and ready to eat. |
| *Githeri* | Mixture of maize and beans | Both mixed together and boiled until ready to eat. |
| Waste water |  | Water from a channel carrying liquid and solid waste, including rainwater, floodwater, and sewage |
| Drinking water |  | Water supplied by the municipal county including both “legal” and “illegal” connections. Water may be accessed through: piped water into compounds (including flexible pipes); public taps/standpoints (a formally designated water station in the community, provided by the government, or managed by someone in the community); or water vendors/trucks. All drinking water samples were collected directly from the source and not from household storage containers. |
