# Supplementary figures and images for: Molecular detection and antibiotic resistance of diarrheagenic Escherichia coli from street food and water in mukuru slums, Nairobi County
Source: PLoS One. 2026 Jan 28;21(1):e0340081. doi: 10.1371/journal.pone.0340081 (PMC12851472; doi:10.1371/journal.pone.0340081)

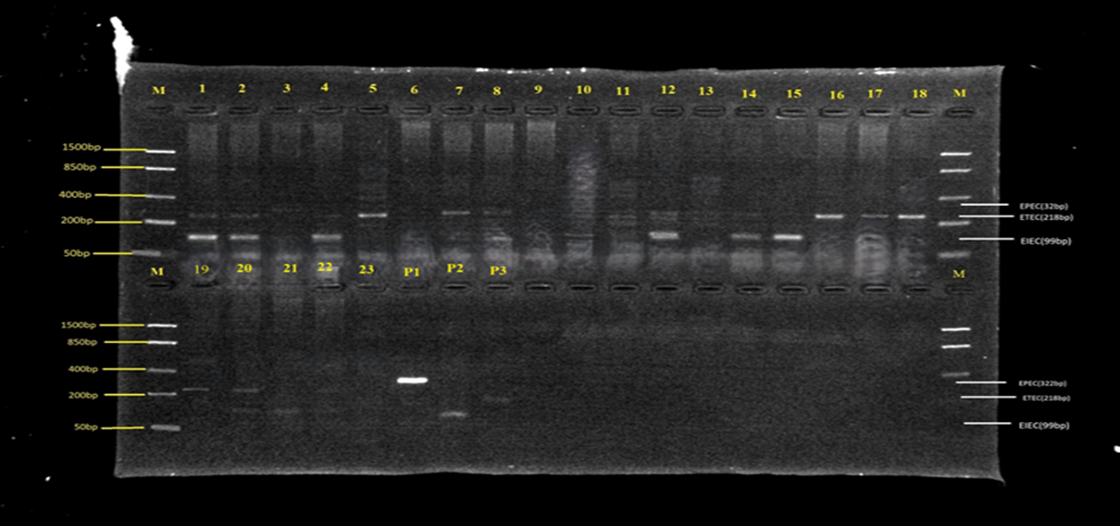

Supplement: S1 Fig — (ZIP) [file pone.0340081.s001.zip › Updated Supporting Information/S2 Multiplex Image (1) (2) (3).tif]

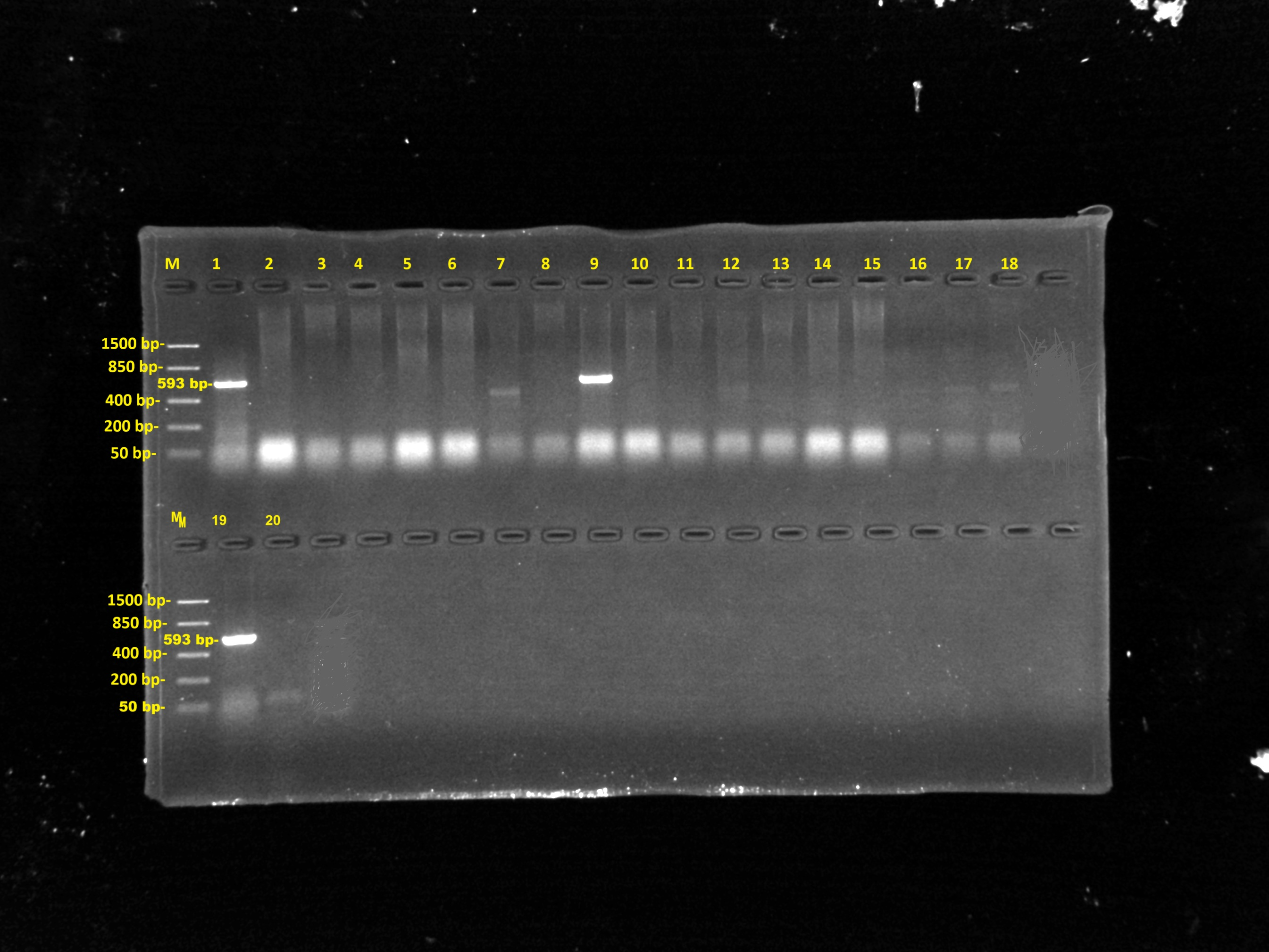

Supplement: S1 Fig — (ZIP) [file pone.0340081.s001.zip › Updated Supporting Information/S3 ESBL CTXM _Gel image (1).tif]
