# Supplementary material for: Molecular detection and antibiotic resistance of diarrheagenic Escherichia coli from street food and water in mukuru slums, Nairobi County
Source: PLoS One. 2026 Jan 28;21(1):e0340081. doi: 10.1371/journal.pone.0340081 (PMC12851472; doi:10.1371/journal.pone.0340081)
Supplement: Attachment — Submitted filename: PONE-D-24-59070_R1.pdf [file pone.0340081.s004.pdf]

PLOS ONE

Molecular Detection and Antibiotic Resistance of Diarrheagenic Escherichia coli from Street food and Water in Mukuru slums, Nairobi County.

--Manuscript Draft--

**Manuscript Number:**	PONE-D-24-59070R1
**Article Type:**	Research Article
**Full Title:**	Molecular Detection and Antibiotic Resistance of Diarrheagenic Escherichia coli<br><br>from Street food and Water in Mukuru slums, Nairobi County.
**Short Title:**	Molecular Detection and AMR of DEC from food and Water in Nairobi Slums
**Corresponding Author:**	Sheillah Nabifwo Mundalo, Msc Microbiology<br>Kenya Medical Research Institute<br>NAIROBI, KENYA
**Keywords:**	Antibiotic resistance, wastewater, street food, ESBL
**Abstract:**	Globally, diarrheal diseases account for 550 million cases of foodborne illnesses annually. In Kenya, Escherichia coli (E. coli) infections from contaminated food and water pose a serious health concern, especially in settings with poor sanitation and hygiene practices This study examined the genetic characteristics and antimicrobial resistance profiles of diarrheagenic E. coli (DEC) recovered from street foods and water from Mukuru informal settlements, Nairobi. Between September and December 2023, 384 (each 77) samples of street foods (Mandazi, githeri, French fries), wastewater, and drinking water were collected and E. coli isolation performed through microbiological culture and antibiotic susceptibility testing done using Kirby-Bauer disc diffusion method. Conventional Polymerase chain reaction (PCR) was used to screen for six DEC and extended-spectrum beta-lactamase (ESBL) resistance genes. Data was statistically analyzed using descriptive methods to determine the prevalence and inferential test using chi-square to assess associations E. coli was isolated in 16% (62/384) of the samples, with 77.4% (48/62) of the isolated E. coli being DEC. Majority (64.6%, 31/48) of DEC isolates were recovered from wastewater followed by drinking water (22.9%, 11/48), githeri (8.3%, 4/48), mandazi (4.2, 2/48), and none from french fries. The most common pathotypes were; ETEC 69% (33/48), STEC 52.1% (25/48), EIEC 50% (24/48)), EPEC 10% (5/48), and EAEC 6% (3/48) Of the 48 DEC isolates, 30 were hybrid isolates Overall, the E. coli isolates were highly resistant to tetracycline (77.4%), trimethoprim-sulfamethoxazole (71.0%), ampicillin (59.7%) and least resistant to kanamycin (16.1%); chloramphenicol (8.1%) and amoxicillin + clavulanic acid (4.8%). A huge proportion (72.6%, 45/62) of the isolated E. coli were multidrug-resistant (MDR). Of the 45 MDR isolates, 60% (27) were from wastewater, 28.9% (13) from drinking water, 4.4% (2) from githeri, 4.4% (2) from mandazi and 2.2% (1) french fries 2.2%. ESBL genes bla-TEM and bla CTX-M were detected in 12.9% and 3.2% of the E. coli isolates. isolates. The high prevalence of MDR in the E .coli isolates recovered from environmental compartments and food is a huge public health risk to the population in these settings. The detection of E. coli indicates fecal contamination underscoring the need to improve water, and sanitation infrastructure in urban slums.<br>Keywords: Antibiotic resistance, wastewater, street food, ESBL
**Order of Authors:**	Sheillah Mundalo, Msc Microbiology
	Regina Ntabo, PhD
	Kelvin Kering, PhD in Medical Microbiology
	Real Too, PhD
	Kevin Kariuki, Msc Biotechnology
	Diana Imoli, Msc Microbiology
	Brian Silantoi, Msc. Medical Microbiology
	Susan Kavai, PhD
	Evans Kiptanui

Powered by Editorial Manager® and ProduXion Manager® from Aries Systems Corporation

	Samuel Kariuki, PhD
	Cecilia Mbae, PhD
**Response to Reviewers:**	Dear Reviewers,thank you for the thorough review you have done on my paper.It means alot to my career growth.i have attcahed all the responses in the response letter that has been attached.
**Additional Information:**	
**Question**	**Response**
**Financial Disclosure**<br><br>Enter a financial disclosure statement that describes the sources of funding for the work included in this submission. Review the submission guidelines for detailed requirements. View published research articles from *PLOS ONE* for specific examples.<br><br>This statement is required for submission and **will appear in the published article** if the submission is accepted. Please make sure it is accurate.<br><br>**Funded studies**<br>Enter a statement with the following details:<br>• Initials of the authors who received each award<br>• Grant numbers awarded to each author<br>• The full name of each funder<br>• URL of each funder website<br>• Did the sponsors or funders play any role in the study design, data collection and analysis, decision to publish, or preparation of the manuscript?<br><br>Did you receive funding for this work?	The author(s) received no specific funding for this work.
**Competing Interests**<br><br>Use the instructions below to enter a competing interest statement for this submission. On behalf of all authors, disclose any competing interests that could be perceived to bias this work—acknowledging all financial support and any other relevant financial or non-financial competing interests.	The authors have declared that no competing interests exist.

This statement is required for submission and **will appear in the published article** if the submission is accepted. Please make sure it is accurate and that any funding sources listed in your Funding Information later in the submission form are also declared in your Financial Disclosure statement.<br><br>View published research articles from *PLOS ONE* for specific examples.<br><br>**NO authors have competing interests**<br><br>Enter: *The authors have declared that no competing interests exist.*<br><br>**Authors with competing interests**<br><br>Enter competing interest details beginning with this statement:<br><br>*I have read the journal's policy and the authors of this manuscript have the following competing interests: [insert competing interests here]*<br><br>* typeset	
**Ethics Statement**<br><br>Enter an ethics statement for this submission. This statement is required if the study involved:<br><br>• Human participants<br>• Human specimens or tissue<br>• Vertebrate animals or cephalopods<br>• Vertebrate embryos or tissues<br>• Field research<br><br>Write "N/A" if the submission does not require an ethics statement.<br><br>General guidance is provided below. Consult the submission guidelines for detailed instructions. **Make sure that all information entered here is included in the Methods section of the manuscript.**	Ethical approval was obtained from the institutional review board at Kenya Medical Research Institute; Scientific and Ethics Review Unit (KEMRI/SERU/CMR/P00240/4770).

Powered by Editorial Manager® and ProduXion Manager® from Aries Systems Corporation

**Format for specific study types**<br><br>**Human Subject Research (involving human participants and/or tissue)**<br>• Give the name of the institutional review board or ethics committee that approved the study<br>• Include the approval number and/or a statement indicating approval of this research<br>• Indicate the form of consent obtained (written/oral) or the reason that consent was not obtained (e.g. the data were analyzed anonymously)<br><br>**Animal Research (involving vertebrate animals, embryos or tissues)**<br>• Provide the name of the Institutional Animal Care and Use Committee (IACUC) or other relevant ethics board that reviewed the study protocol, and indicate whether they approved this research or granted a formal waiver of ethical approval<br>• Include an approval number if one was obtained<br>• If the study involved *non-human primates*, add *additional details* about animal welfare and steps taken to ameliorate suffering<br>• If anesthesia, euthanasia, or any kind of animal sacrifice is part of the study, include briefly which substances and/or methods were applied<br><br>**Field Research**<br><br>Include the following details if this study involves the collection of plant, animal, or other materials from a natural setting:<br>• Field permit number<br>• Name of the institution or relevant body that granted permission	
**Data Availability**<br><br>Authors are required to make all data underlying the findings described fully available, without restriction, and from the time of publication. PLOS allows rare exceptions to address legal and ethical concerns. See the PLOS Data Policy and FAQ for detailed information.	Yes - all data are fully available without restriction

A Data Availability Statement describing where the data can be found is required at submission. Your answers to this question constitute the Data Availability Statement and **will be published in the article**, if accepted.<br><br>**Important:** Stating 'data available on request from the author' is not sufficient. If your data are only available upon request, select 'No' for the first question and explain your exceptional situation in the text box.<br><br>Do the authors confirm that all data underlying the findings described in their manuscript are fully available without restriction?	
**Describe where the data may be found in full sentences. If you are copying our sample text, replace any instances of XXX with the appropriate details.**<br><br>• If the data are **held or will be held in a public repository**, include URLs, accession numbers or DOIs. If this information will only be available after acceptance, indicate this by ticking the box below. For example: *All XXX files are available from the XXX database (accession number(s) XXX, XXX.*).<br>• If the data are all contained **within the manuscript and/or Supporting Information files**, enter the following: *All relevant data are within the manuscript and its Supporting Information files.*<br>• If neither of these applies but you are able to provide **details of access elsewhere**, with or without limitations, please do so. For example:<br><br>*Data cannot be shared publicly because of [XXX]. Data are available from the XXX Institutional Data Access / Ethics Committee (contact via XXX) for researchers who meet the criteria for access to confidential data.*<br><br>*The data underlying the results presented in the study are available*	All relevant data neede to replicate the analysis are within the manuscript and its supportinng information files

from (include the name of the third party and contact information or URL).<br>• This text is appropriate if the data are owned by a third party and authors do not have permission to share the data.<br><br>* typeset	
Additional data availability information:	Tick here if the URLs/accession numbers/DOIs will be available only after acceptance of the manuscript for publication so that we can ensure their inclusion before publication.; Tick here if your circumstances are not covered by the questions above and you need the journal’s help to make your data available.

Manuscript

Click here to access/download;Manuscript;Manuscript docx...... fin.docx

Molecular Detection and Antibiotic Resistance of Diarrheagenic *Escherichia coli* from Street Food and Water in Mukuru slums, Nairobi County.

Sheillah Mundalo[1,2*], Regina Ntabo[2], Kelvin Kering[1], Rael Too[1], Kevin Kariuki[1], Diana Imoli[1], Brian Silantoi[1], Evans Kiptanui[1], Susan Kavai[1], Samuel Kariuki[1] Cecilia Mbae[1]

[1] Centre for Microbiology Research, Kenya Medical Research Institute, Nairobi, Kenya.

[2] Department of Biotechnology, Biochemistry and Microbiology, Kenyatta University, Nairobi, Kenya.

*Correspondence: smundalo@kemri.go.ke

Abstract

Globally, diarrheal diseases account for 550 million cases of foodborne illnesses annually. In Kenya, *Escherichia coli* (*E. coli*) infections from contaminated food and water pose a serious health concern, especially in settings with poor sanitation and hygiene practices This study examined the genetic characteristics and antimicrobial resistance profiles of diarrheagenic *E. coli* (DEC) recovered from street foods and water from Mukuru informal settlements, Nairobi. Between September and December 2023, 384 (each 77) samples of street foods (*Mandazi, githeri, French fries*), wastewater, and drinking water were collected and *E. coli* isolation performed through microbiological culture and antibiotic susceptibility testing done using Kirby-Bauer disc diffusion method. Conventional Polymerase chain reaction (PCR) was used to screen for six DEC and extended-spectrum beta-lactamase (ESBL) resistance genes. Data was statistically analyzed using descriptive methods to determine the prevalence and inferential test using chi-square to assess associations *E. coli* was isolated in 16% (62/384) of the samples, with 77.4% (48/62) of the isolated *E. coli* being DEC. Majority (64.6%, 31/48) of DEC isolates were recovered from wastewater followed by drinking water (22.9%, 11/48), *githeri (*8.3%, 4/48), *mandazi* (4.2, 2/48), and none from *french fries*. The most common pathotypes were; ETEC 69% (33/48), STEC 52.1%

(25/48), EIEC 50% (24/48)), EPEC 10% (5/48), and EAEC 6% (3/48) Of the 48 DEC isolates, 30 were hybrid isolates Overall, the *E. coli* isolates were highly resistant to tetracycline (77.4%), trimethoprim-sulfamethoxazole (71.0%), ampicillin (59.7%) and least resistant to kanamycin (16.1%); chloramphenicol (8.1%) and amoxicillin + clavulanic acid (4.8%). A huge proportion (72.6%, 45/62) of the isolated *E. coli* were multidrug-resistant (MDR). Of the 45 MDR isolates, 60% (27) were from wastewater, 28.9% (13) from drinking water, 4.4% (2) from githeri, 4.4% (2) from mandazi and 2.2% (1) french fries 2.2%. ESBL genes *bla*-$_{\text{TEM}}$ and *bla* $_{\text{CTX-M}}$ were detected in 12.9% and 3.2% of the *E. coli* isolates. isolates. The high prevalence of MDR in the *E .coli* isolates recovered from environmental compartments and food is a huge public health risk to the population in these settings. The detection of *E. coli* indicates fecal contamination underscoring the need to improve water, and sanitation infrastructure in urban slums.

Keywords: Antibiotic resistance, wastewater, street food, ESBL

Introduction

Food and water safety is an important component of public health and economic prosperity. A large proportion of contaminated food results in diarrheal diseases, with diarrheal disease agents accounting for 550 million of the 600 million cases of foodborne illness (1). Each year, an estimated 600 million people fall ill and 420,000 people die from unsafe food (2). Global Burden of Foodborne Diseases report estimated that 111 million illnesses and 63,000 deaths are caused by diarrhoeagenic *E. coli* globally each year (3). Low- and middle-income countries (LMICs) bear the highest burden of diarrheal disease, predominantly caused by *E. coli* (4)**.** The sale of ready-to-eat street food represents an important source of income in many developing countries, however, studies have highlighted street foods as a major public health risk (5).

In Kenya, diarrheal diseases rank as the third most significant cause of pediatric morbidity and mortality, after malaria and tuberculosis [6]. *E. coli* is a major contributor to ill health, including neonatal meningitis, urinary tract infections (UTIs), pyelonephritis, cystitis, septicemia, and

traveler's diarrhea (7). Exposure to *E. coli* most commonly occurs through ingesting contaminated food and water. Previous studies have indicated that pathogenic strains of *E. coli* develop from commensal/naïve bacteria by acquiring specific virulence genes and operons either on the chromosome or through extra-chromosomal elements (8). Diarrheagenic *E. coli* is categorized into five distinct pathotypes based on their clinical manifestation' phenotypic traits, epidemiological evidence, and virulence factors specific to each pathotype (9). They include; Enteroaggregative (EAEC), Shiga-toxigenic (STEC), Enteroinvasive (EIEC), Enteropathogenic (EPEC) and Enterotoxigenic (ETEC). Antibiotic treatment is the primary method for preventing and managing bacterial infections. However, the growing antibiotic resistance among bacterial pathogens transmitted through food poses a major challenge, leading to increased mortality and morbidity from foodborne illnesses resulting in high socioeconomic costs (10). Different studies conducted on different items of ready-to-eat foods vended on the street in low-income countries, revealed different levels of contamination and the majority of foodborne bacterial pathogens were resistant to commonly prescribed antibiotics (11). The emergence of antimicrobial resistance has led to challenges in the management of infectious diseases that affect both animals and humans (12). The World Health Organization has ranked *E. coli* as the second most important pathogen globally that is resistant to antimicrobials(13). The spread of antimicrobial resistance in pathogenic strains of *E. coli* is critical because infections caused by these strains are often harder to treat, resulting in increased severity and duration of infection (14). One important group of antimicrobial-resistant *E. coli* is the ESBL-producing *E. coli* (15). Since bacteria are highly prone to exchanging genetic material, these resistance genes are easily disseminated between bacteria (16).

The rise of multidrug-resistant *E. coli* strains poses a significant public health challenge and complicates the management of serious infections (17). In Kenya, there is evidence of multidrug-

resistant *E. coli* isolated from clinical and environmental samples (18). The prevalence of diarrheagenic *E. coli* (DEC) and its resistance profiles from street food and water is not well known especially in low-resource settings such as slums. The present study sought to determine the occurrence, distribution and AMR profiles of DEC pathotypes in street foods and water samples from Mukuru informal settlement. This study investigated multidrug-resistant *E. coli* and the prevalence of pathogenic *E. coli* isolated from street food and water samples in the Mukuru informal settlement in Nairobi.

Materials and Methods

Study site and design

This was a cross-sectional study where samples were randomly collected from food vendors and water samples from boreholes in Mukuru Informal settlement. Mukuru is located 20km East of Nairobi city center. With a population of approximately 150,000, it covers an area of approximately 2 km^2 (population density, 75,000/km^2)(19). Only 1% of residents in Mukuru have access to a private or individual water source. There is an average of 234 households per public water tap in Mukuru and the households are water insecure, since they have access to 1.8 cubic meters of water per month, while the international recommendation for health and hygiene is 4 cubic meters per month (20).

Sample collection

We standardized the SaniPath protocol (21) that has been applied in exposure assessment to obtain our samples **(S1)** (21). After obtaining verbal consent, we collected the street food vendors prepared as a single serving and aseptically poured/placed the food into a 500 mL Whirl-Pak bag.

Drinking water was collected from communal municipal water points into a 500 mL Whirl-Pak while wastewater sampling points were randomly selected based on areas where human interaction was most frequent. The study employed the Fischer formula $n= Z^2PQ/d^2$ to determine a sample size of 384 where n is the estimated sample size, Z is the Z-score corresponding to the desired confidence level (1.96 for a 95% confidence level), P is the estimated proportion (0.50 or 50% in this case since no previous estimate is available), Q is the complementary probability to P, which is equal to 1 -P and d is degree of accuracy (0.05 at 95% confidence level)

Samples were then aseptically transported to the KEMRI microbiology laboratory in cooler boxes and processed within six hours of sample collection.

Laboratory Analysis

Samples from street foods were rinsed in distilled water and then a loopful of the 10 mL of rinse solution was cultured in Chromocult® Coliform Agar (Millipore at # ISO 9308-1). Water samples were cultured directly in Chromocult® Coliform Agar. After 18 hours of incubation, *E. coli* suspects (blue colonies) were sub-cultured on Muller Hilton agar and incubated for 18hrs. *E. coli* identification was done using analytical profile index (API) 20E strips (bioMerieux, Inc., Durham, North Carolina, United States).

DNA was extracted from pure *E. coli* colonies using the boiling method (22). A loop full of the colony was suspended in 200 µl of PCR water in an Eppendorf tube and heated at 95°C for 8 minutes on a heating block. Centrifugation was then done at 15,000g for 5 minutes at 25°C. The DNA-containing supernatant was aliquoted to a sterile Eppendorf tube.

Detection of *E. coli* pathotypes

Multiplex polymerase chain reaction assays were used for the detection of five types of DEC as previously described (23). Multiplex was done in EPEC, ETEC, and EIEC while singleplex was

done on EAEC and STEC. The targeted genes for the pathogenic *E. coli* strains (**Table 1**) included ETEC (*lt* encoding heat-labile enterotoxins), STEC (stx_1 encoding Shiga-toxin), EPEC (*bfp* encoding bundle forming pili), EAEC (*aggR* encoding transcription regulator for aggregative adherence fimbria I), and EIEC (*ipaH* encoding invasion plasmid antigen H). All *E. coli* isolates and positive controls for each of the genes tested had their DNA extracted using the boiling method as previously described (22). PCR water was used as a negative control. The cycling conditions for all the PCR amplifications of pathotypes genes were as follows: 94˚C for 5 minutes for initial denaturation and 30 cycles of 95˚C for 30 seconds (denaturation), annealing at 62˚C for 1 minute (for other primers) and 72˚C for 1 minute 30 seconds (extension), followed by 72˚C for 10 minutes (final extension); performed in 0.2 mL Eppendorf PCR tubes in a thermal cycler (MJ Research PTC-200). Amplified PCR products were separated and visualized by electrophoresis using 1% agarose tris-acetate-EDTA gel containing 0.5 µg EtBr staining. A 100–1,000 bp DNA ladder (Fermenters) was used to determine molecular weight.

Table 1: *Escherichia coli* pathotypes genes analyzed and the primers

Genes	Primer sequence (5’-3’)	Product size (Bp)	Annealing temp	Reference
bfpA	F: AATGGTGCTTGCGCTTGCTGC	324	60	(24)
	R: CCGCTTTATCCAACCTGGTA			
stx1	F: AGTTAATGTGGTGGCGAA	817	58	(25)

Genes	Primer sequence (5’-3’)	Product size (Bp)	Annealing temp	Reference
	R:GACTTGCCGCTTCCAT			
aggR	F: GTATACACAAAGAAGGAAGC	254	58	(25)
	R: ACAGAATCGTCAGCATCAGC			
LT	F: GCACACGGAGCTCCTCAGTC	218	60	(25)
	R: TCCTTCATCCTTTCAATGGCTTT			
IpaH	F- AGGTCGCTGCATGGCTGGAA<br>R- CACGGTCCTCACAGCTCTCA	99	61	(26)

Antibiotic susceptibility testing

The antimicrobial sensitivity profiles of all the isolated *E. coli* isolates were performed using the Kirby Bauer disk diffusion method against 12 antibiotics according to the Clinical and Laboratory Standards Institute guidelines (27). There were seven drug classes tested: Beta-lactam, Amoxicillin + Clavulanic acid AMC (30 µg).;ampicillin AMP (10 µg).; ceftriaxone CRO (30 µg). ; ceftazidime CAZ (30µg), cefotaxime CTX(30µg), cefpodoximine CPD(10µg) Aminoglycosides (gentamicin CN(10µg), Quinolones (nalidixic acid NA(30µg); ciprofloxacin CIP(5µg), Sulfonamides (trimethoprim-sulfamethoxazole STX (25µg), Macrolides (azithromycin(15µg), Phenicols (chloramphenicol CHL (30 µg). Tetracyclines (tetracycline TCY (30 µg) guidelines were provided

by the Clinical and Laboratory Standards Institute (CLSI) in 2023. *E. coli* ATCC 25922 was used as a positive control. MDR was defined as resistance to at least one antibiotic from different antibiotic classes.

The zones of inhibitions were measured and interpreted using CLSI M100. Isolates that were resistant to 3rd cephalosporin were scored as ESBLs (Extended Spectrum Beta-lactamase).

Screening for extended-spectrum β-lactamase

Phenotypic methods were used for further confirmation of β-lactamase production for all the isolates showing resistance to 3rd generation cephalosporin, namely ceftazidime, cefotaxime, and ceftriaxone. A combination of clavulanic acid (10µg) and ceftazidime (30 µg) was used. Both discs were placed on Muller Hinton agar plates which were earlier swabbed by respective cultures and incubated for 24 hours at 37°C. More than 5mm increase in the zone diameter for ceftazidime-clavulanic acid was considered positive ESBL production.

ESBL detection and ESBL-associated genes ($bla_{\text{CTX-M}}$, bla_{TEM}, and bla_{SHV}) were detected using conventional PCR and previously described primers. **Table 2** (19)

Table 2. Resistance genes analyzed and the primers used

Target Gene	Primer sequence (5'-3')	Product size bp	Annealing temp (°C)	References
bla_{TEM}	F: GCGGA ACCCCTA TTTG	1120	62	(25)
	R: TCTAAAGTATATATGAGTAAACTTGG TCTGAC			

Target Gene	Primer sequence (5’-3’)	Product size bp	Annealing temp (°C)	References
bla$_{\text{CTX-M}}$	F: ATGTGCAGGACCAGTAARGTATGGC	593	58	(25)
	R:TGGGTRAARTARGTSACCAGAAYCAGCGG			
bla$_{\text{SHV}}$	F:TTCGCCTGTGTATTATCTCTCCTG	854	58	(25)
	R:TTAGCGTTGCCAGTGTYTCG			

Data Analysis

Descriptive analysis was used to show the distribution of *E. coli* and *E. coli* pathotypes per sample. Inferential analysis, using chi-square statistics, was applied to assess associations. Antimicrobial-resistant patterns were interpreted and analyzed using the M 100 Clinical and Laboratory Standards Institute (CLSI 2022) and ~~the Antimicrobial resistant patterns were~~ analyzed using WHONET 2024 https://whonet.org/

Ethical Considerations

Ethical approval was obtained from the institutional review board at Kenya Medical Research Institute; Scientific and Ethics Review Unit (KEMRI/SERU/CMR/P00240/4770). Verbal informed consent was sought from the street food vendors before buying the street foods.

Results

Distribution of *E. coli* among the samples (Street food and water samples)

A total of 384 samples (wastewater, drinking water, fries, githeri and mandazi) ~~each~~ ~~77,~~ were collected and analyzed. Out of the 384 samples, a total of 62 *E. coli* isolates were identified. Majority (58.1%, ~~36~~) of the ~~62 *E. coli*~~ isolates~~,~~ were detected in wastewater samples, while 25.8% (~~16~~) were isolated from drinking water, 9.7% (~~6~~) from githeri, 4.8% (~~3~~) from mandazi and 1.6% (~~1~~) from french fries. (**Table 3**).

Distribution of Pathogenic *E. coli* per sample

Genomic DNA was extracted, and PCR was done from the 62 isolates of *E. coli* to identify the five *E. coli* pathotypes. Out of these, commensal *E. coli* was 22.6% (14/62) while pathogenic was 77.4% (48/62) which were positive for at least one pathotype. Wastewater had the highest proportion 64.6.3% (31/48) of pathogenic *E. coli*, followed by drinking water 22.9% (11/48), githeri 8.3% (4/48), and mandazi 4.2% (2/48) (Table 3).

T[illegible]e 3: Distribution of *E. coli* and *E. coli* pathotypes recovered from different samples collected

**Sample Type**	**Samples positive for *E.coli***	**Samples positive for E.coli pathotypes**
Wastewater	36 58.1%	31 64.6%

Drinking water	16 25.8%	11 22.9%
French fries	1 1.6%	0
Mandazi	3 4.8%	2 4.2%
Githeri	6 9.%	4 8.3%

Distribution of *E. coli* pathotypes recovered from different samples collected in Mukuru Informal settlement.

The overall distribution of the pathotypes were as follows; ETEC 69%(33/48), STEC 52.1%(25/48), EIEC 50%(24/48),EPEC 10%(5/48) and EAEC6%(3/48). As shown in **Fig. 1, (Overall prevalence of *E. coli* pathotypes) .** ETEC was the most prevalent pathotype across all samples while hybrid pathotypes were detected in 30 isolates. Wastewater isolate had all the five pathotypes with ETEC being the most predominant pathotype. Drinking water and *mandazi* had four pathotypes detected while *githeri* had three pathotypes, as shown in **Fig 2. (Prevalence of *E. coli* pathotypes per sample)**

Hybrid Pathogenic Types Detected

Of 48 pathotypes identified, 30 isolates had hybrid pathogenic types that formed two, three and four pathotypes. The most diverse hybrid pathotypes were found in *E. coli* pathotypes from wastewater, as shown in **Fig. 3. (Hybrid distribution per sample).** The hybrid strains in githeri and mandazi samples were relatively low compared to other sample types. ETEC, STEC, and ETEC, EIEC were the most prevalent hybrid combinations with a prevalence of 23% (7/30) each as shown in **Fig. 4. Hybrid distribution of the pathotypes**. ETEC, STEC, EIEC had a prevalence of 20% (6/30). Among the hybrids, ETEC was the most prevalent pathotype.

Antimicrobial Resistance Profiles

The highest overall resistance of the 62 *E. coli* isolates was observed in tetracyclines (77.4%), sulfonamides (71.0%), and ampicillin (59.7%). Moderate resistance was observed in cefotaxime (50.0%), ciprofloxacin (41.9%), and azithromycin (37.1%). Low resistance rates were observed in kanamycin (16.1%), chloramphenicol (8.1%) and amoxicillin-clavulanic acid (4.8%) (Table 4).

Table 4: Overall Antibiotic Resistance of *E.coli* isolated from the samples (n/62)

Drug	Drinking water		Fries		Githeri		Mandazi		Waste water		Total	
	n	%	n	%	n	%	n	%	n	%	n	%
TCY	13	21.0%	1	1.6%	2	3.2%	3	4.8%	29	46.8%	48	77.4%
SXT	11	17.7%	1	1.6%	3	4.8%	3	4.8%	26	41.9%	44	71.0%
AMP	11	17.7%	1	1.6%	2	3.2%	2	3.2%	21	33.9%	37	59.7%
CTX	8	12.9%			2	3.2%			21	33.9%	31	50.0%
CIP	10	16.1%							16	25.8%	26	41.9%
AZM	6	9.7%			3	4.8%	1	1.6%	13	21.0%	23	37.1%
CRO	7	11.3%			2	3.2%			13	21.0%	22	35.5%
CPD	6	9.7%			1	1.6%	1	1.6%	12	19.4%	20	32.3%
GEN	6	9.7%			1	1.6%	1	1.6%	12	19.4%	20	32.3%
CAZ	5	8.1%			2	3.2%			11	17.7%	18	29.0%
NAL	7	11.3%							7	11.3%	14	22.6%
KAN	3	4.8%					1	1.6%	6	9.7%	10	16.1%
CHL	2	3.2%							3	4.8%	5	8.1%
AMC									3	4.8%	3	4.8%

KEY: TCY -tetracycline, SXT -trimethoprim-sulfamethoxazole, AMP–ampicillin, CTX-cefotaxime, CIP –ciprofloxacin, AZM –azithromycin, CRO –ceftriaxone, CPD-cefpodoxime, GEN-gentamicin, CAZ –ceftazidime, NAL-nalidixic acid, KAN-kanamycin, CHL-chloramphenicol, AMC-amoxicillin-clavulanic acid

DEC Resistance Patterns to Antibiotics

All 48 pathotypes exhibited varying levels of resistance to the antibiotics. ETEC exhibited high resistance rates to all the antibiotics compared to the other pathotypes, as shown in **Figure 5. (Drug resistance per pathotype**).ETEC exhibited high resistance to tetracycline (47.9%), trimethoprim-sulfamethoxazole (41.7%), cefotaxime (39.6%), and ampicillin (37.5%). STEC showed resistance

to trimethoprim-sulfamethoxazole (45.8%), tetracycline (41.7%) ampicillin (35.4%). EAEC exhibited low resistance to ampicillin (6.3%), trimethoprim-sulfamethoxazole (6.3%), and tetracycline (6.3%); EPEC exhibited resistance to ampicillin (10.4%), trimethoprim-sulfamethoxazole (8.3%), and tetracycline (8.3%). EIEC had varying resistance rates to ampicillin (22.9%), trimethoprim-sulfamethoxazole (29.2%), and tetracycline (35.42%).

EIEC did not exhibit any resistance to amoxicillin-clavulanic acid, while EAEC did not exhibit any resistance to azithromycin, chloramphenicol, and nalidixic acid. EPEC did not show any resistance to amoxicillin-clavulanic acid and chloramphenicol

Association between Sample Type and Antibiotic Resistance

Inferential analysis, using chi-square statistics, was applied to assess associations between sample type and antibiotics. *E. coli* isolates from wastewater had resistance across all the tested antibiotics, with significant resistance to ampicillin (21/62 isolates), cefotaxime (21/62 isolates), and tetracycline (29/62 isolates). *E. coli* isolates from drinking water also had high resistance to tetracycline (13/62), ampicillin (11/62), trimethoprim-sulfamethoxazole (11/62), and ciprofloxacin (10/62). *E. coli* isolates from *Mandazi* and *githeri* had lower resistance overall but still exhibited resistance to beta-lactams, tetracycline, and sulfonamides. There was a significant difference in resistance to ciprofloxacin ($p = 0.030$).

Table 5: Association between sample type and Antibiotics

**Sample type**	**Antibiotic Resistance**													
	**AMC**	**AMP**	**AZM**	**CTX**	**CPD**	**CAZ**	**CRO**	**CHL**	**CIP**	**GEN**	**KAN**	**NAL**	**TCY**	**SXT**
Drinking water		11	6	8	6	5	7	2	10	6	3	7	13	11

French fries		1											1	1
Githeri		2	3	2	1	2	2			1			2	3
Mandazi		2	1		1					1	1		3	3
Wastewater	3	21	13	21	12	11	13	3	16	12	6	7	29	26
**P-Values**	0.533	0.849	0.937	0.359	0.699	0.608	0.619	0.956	**0.030**	0.731	0.582	0.059	0.172	0.561

KEY: TCY -tetracycline, SXT -trimethoprim-sulfamethoxazole, AMP–ampicillin, CTX-cefotaxime, CIP –ciprofloxacin, AZM –azithromycin, CRO –ceftriaxone, CPD-cefpodoxime, GEN-gentamicin, CAZ –ceftazidime, NAL-nalidixic acid, KAN-kanamycin, CHL-chloramphenicol, AMC-amoxicillin-clavulanic acid

Multidrug resistance Patterns

Distribution of MDR *E. coli* (n = 45) isolated from different samples

Of the 45 MDR isolates, 60% (27) were from wastewater, 28.9% (13) from drinking water, 4.4% (2) from githeri, 4.4% (2) from mandazi and 2.2% (1) french fries 2.2%(1).**Fig. 6 (Prevalence of MDR *E. coli* isolates from different samples**). The frequency of resistance to 3, 4, 5, 6, and 7 classes of antimicrobial agents were 17.8% (8/45), 22.2% (10/45), 24.4% (10/45), 24.4% (10/45), 11.1% (5/45), respectively. **Figure 7 (MDR *E. coli* according to the number of drug class resistance)**

Distribution of ESBL genes Per Sample

Of the 62 *E. coli* isolates, 12.9% (8) carried *bla*$_{TEM}$ gene, of these, six were detected in wastewater while two in drinking water. The *bla*$_{CTXM}$ gene was detected in 3.2% (2) of the *E. coli* isolates accounting for one in drinking water and one in wastewater. The *bla*$_{TEM}$ gene was detected in

multiple pathotypes, including ETEC, EAEC, STEC, EIEC, and EPEC. The *bla*$_{CTXM}$ gene was found in isolate that was not pathogenic and a hybrid isolate of ETEC, EPEC, and EIEC as shown in table 6

Table 6: Distribution of ESBL genes Per Sample

Sample Type	*E. coli* Pathotype	ESBL Gene
Wastewater	ETEC, EAEC	TEM
Wastewater	EPEC, STEC, ETEC	TEM
Wastewater	ETEC, STEC, EIEC	TEM
Wastewater	ETEC, STEC, EIEC	TEM
Wastewater	ETEC, STEC	TEM
Wastewater	ETEC, STEC, EPEC	TEM
Drinking water	ETEC, STEC	TEM
Drinking water	STEC	TEM
Drinking water	Negative	CTX-M
Wastewater	ETEC, EPEC, EIEC	CTX-M

.

Discussion

Foodborne diseases caused by *E. coli* contamination are responsible for a huge public health challenge globally especially in low- and middle-income countries LMICs (1). Understanding the role of environmental reservoirs in disseminating diarrheagenic *E. coli* is crucial for mitigation strategies. This study was conducted in an urban informal settlement characterized by high population, dilapidated water, sanitation, and hygiene (WASH) infrastructure; therefore, most residents use common water points for drinking water. Street foods are very popular within these areas because they are relatively inexpensive, convenient, and easily accessible on the sidewalk (28). These conditions could contribute to a high burden of diarrheal morbidity within the population especially among children (19).

The detection of *E. coli* pathotypes in water and food samples highlight the potential public health risks associated with these samples (29) in this setting. Wastewater had a high *E.coli* isolation rate compared to drinking water: similar findings were observed in a study conducted in Peru that showed drinking water samples had a lower prevalence of *E. coli* contamination, yet still posed a health concern (30) according to WHO standards, 2023. *French fries* showed minimal contamination, with only 1% testing positive, however, this is concerning as these are ready to eat foods and could easily result in foodborne infections. The minimal detection of *E. coli* in French fries might be attributed to the frying process, which involves high temperatures capable of eliminating bacterial pathogens as has been shown previously (31). The contamination could therefore be due to poor handling and post-preparation. Other food items like *mandazi* and *githeri* exhibited moderate contamination levels, likely due to post-cooking handling or cross-contamination during storage, which is in agreement with a previous aligning with a study on food safety practices in informal settings in Addis Ababa (32).

DEC was prevalent in all sample types except *French fries*, with wastewater showing the highest contamination levels (64.6%) and drinking water following at 22.9%. The detection of DEC in street foods like g*itheri* and *mandazi* highlights the likelihood of these ready-to-eat foods to being fecally contaminated during preparation, handling, or storage (33). These findings are comparable to findings observed in Kat River and Fort Beaufort abstraction water where ETEC was found to be the most prevalent pathotype(34). On the contrary, these findings were different from a study conducted in South Africa where diffusely adherent *E. coli* (DAEC) was the only pathotype isolated from wastewater with no other pathotype isolated (35). Another study conducted in Johannesburg, South Africa, highlighted the high prevalence of DEC in environmental water samples with EPEC being the dominant pathotype. Several other pathotypes were also identified, including ETEC, EAEC, EPEC, and EHEC (36).

The high prevalence of ETEC in environmental samples highlighted in this study poses significant public health risks. ETEC is a leading cause of bacterial diarrhea, particularly in children under five and travelers in low-resource settings as reported by a study in Zambia (37). The transmission of ETEC through contaminated water can cause outbreaks, especially where sanitation and hygienic conditions are poor. Its presence in water and food samples suggests widespread fecal contamination, leading to waterborne and foodborne illness and subsequent outbreaks (38).
Our study also detected diverse hybrid pathotype combinations in some samples (Fig 3). These hybrids are significant because they combine virulence factors, potentially leading to more severe disease outcomes (39). The hybrid combination of ETEC and STEC was similar to another study in Sweden (40). Additionally, in this study, some isolates harbored genes encoding for three

different DEC strains. Interestingly, one street food sample had 4 different DEC strains detected ETEC, STEC, EIEC and EAEC. This simultaneous presence in a single food greatly increases the potential for infection and disease transmission among consumers (39). A previous foodborne outbreaks in Germany which spread out to Europe and North America was attributed to contamination of food samples by a hybrid combination of EAEC, STEC (41). All these strains detected are virulent and might contribute to severe diarrhea outbreaks and could pose a potential public health threat to consumers of the street food and the untreated environmental water. The presence of such hybrids in Mukuru could pose a high risk of severe outbreaks, especially given the community's poor sanitation infrastructure.

One of the major challenges in the management of foodborne diseases is the rise of antimicrobial-resistant bacterial strains. The genes conferring resistance can be horizontally transferred from animal-based food sources and environmental reservoirs to the normal human microbiota and pathogens through food and drink chains (42). Antimicrobial resistance in bacteria from food and water is a major threat to global public health (43). From this study, high resistance to ampicillin, sulfamethoxazole and tetracycline was observed across all sample types. The results from this study are similar to a study done in Korogocho, an urban slum in Nairobi, Kenya (44). These drugs are the most frequently used in Kenya (45). This suggests the environmental dissemination of resistant strains from wastewater in urban slums that are significant reservoirs of resistant bacteria (46). These contaminated water sources may serve as vehicles for spreading bacteria and resistance genes to food and drinking water, contributing to the public health burden in areas with poor sanitation (46). Resistance to ciprofloxacin showed a statistically significant association with sample type particularly in wastewater samples, which suggests that such environments might act

as reservoirs for resistant bacteria (46). Our data aligns with findings where wastewater often serves as a hotspot for resistant pathogens due to high antibiotic exposure (47). The high resistance in wastewater observed highlights the need for better waste management systems to prevent the spread of resistant bacteria. Isolates from food samples (*French fries, Githeri, Mandazi*) showed less resistance overall, but vigilance is still required to prevent contamination and spread of AMR through foodborne routes. While investigating *E. coli* from irrigation water and fresh produce in Gauteng, Mosimanegape *et al* found that Aminoglycosides recorded the lowest resistance which is similar to the current study that had Gentamicin and kanamycin recorded low resistance respectively to the antibiotics (48).

The emergence of MDR foodborne bacterial pathogens has recently been regarded as one of the most important concerns in public health and a major challenge in food safety (10). The findings on multidrug resistance among *E. coli* and its pathotypes highlight significant public health concerns. Across all *E.coli* isolates collected, tetracyclines (77.4%) and sulfonamides (71.0%) exhibited the highest resistance levels, consistent with global trends where these antibiotic classes are extensively used in both human and veterinary medicine, contributing to selective pressure on bacteria (49). Lower resistance rates were observed against phenicols and aminoglycosides likely reflecting less frequent usage of these antibiotics similar results as reported by other studies (50). Multidrug resistance was observed across nonpathogenic and pathogenic *E. coli* with isolates from wastewater demonstrating notably high resistance across several antibiotic classes. *E. coli* isolates from drinking water samples also showed moderate to high resistance, with tetracyclines and sulfamethoxazole dominating, highlighting contamination risks from environmental sources. *E. coli* isolates from*French fries* and mandazi displayed alarmingly complete resistance to tetracyclines and sulfonamides underscoring the role of foodborne exposure in spreading resistant

strains. These findings align with studies in sub-Saharan Africa, where resistance to tetracyclines and sulfonamides is also consistently reported as high (51). A study in Kenya by Omulo *et al.* found that over 80% of *E. coli* isolates from both human and environmental sources exhibited resistance to these antibiotic classes, similar to the trends observed in this study. Additionally, studies from Nigeria and Tanzania reported comparable patterns, with tetracyclines and sulfonamides showing the highest resistance rates across environmental and food samples (52)(53). In contrast, resistance to phenicols and aminoglycosides in this study was lower than that reported in India, where resistance to aminoglycosides frequently exceeds 40%, likely due to differences in antibiotic use policies and practices (54).

Among the β-lactam group identified, *bla*$_{\text{TEM}}$ was the most prevalent gene identified. These results were similar to a study conducted in Brazil to isolate virulence genes of *E. coli* isolated from Brazilian farms producers of raw milk fresh cheese that detected within the β-lactam group, was the most prevalent gene detected (55). These results are similar to other studies showing *bla*$_{\text{TEM}}$ and *bla*$_{\text{CTXM}}$ as the most prevalent ESBL genes with no isolation of *bla*$_{\text{SHV.}}$ (56). This occurrence could likely be attributed to the widespread use of third- and fourth-generation cephalosporins. The high resistance to third-generation cephalosporins could lead to the clinical challenges in treating infections, especially with the limited availability of alternative antibiotics in low-resource settings (56).

The high rates of multidrug resistance including the presence of extended-spectrum β-lactamase - producing *E. coli,* have serious implications that suggest widespread environmental contamination with antibiotic resistance genes, potentially due to the overuse or misuse of antibiotics in both human and animal populations.

In conclusion, street food and water samples in Mukuru serve as reservoirs for pathogenic *E. coli,* with the detection of diverse and hybrid pathotypes posing a public health concern, highlighting fecal contamination as a major route for the transmission of waterborne and foodborne pathogens. The presence of hybrid pathotypes, with varied virulence mechanisms and disease outcomes, complicates infections and treatment, particularly with the emergence of multidrug-resistant (MDR) and extended-spectrum beta-lactamase (ESBL)-producing strains resistant to key antibiotics like ampicillin, nalidixic acid, and trimethoprim-sulfamethoxazole. These findings highlight the need for enhanced surveillance and stricter regulations on antibiotic use in agricultural, clinical, and environmental sectors, particularly in low-resource settings. Addressing the One Health dimensions of AMR requires a coordinated approach to mitigate its spread across human, animal, and environmental interfaces.

Acknowledgments

We would like to thank and acknowledge the Kenya Medical Research Institute (KEMRI) and Kenyatta University for infrastructure and laboratory reagents. We also acknowledge the field workers from the Center for Microbiology Research, KEMRI for collecting the samples.

Author Contributions

Conceptualization: Sheillah Mundalo

Lab analysis: Sheillah Mundalo, Diana Imoli, Brian Silantoi

Investigation: Sheillah Mundalo

Project administration: Sheillah Mundalo

Resources: Samuel Kariuki, Kevin Kariuki, Cecilia Mbae ,Susan Kavai, Kelvin Kering

Data analysis: Sheillah Mundalo, Brian Silantoi,Evans

Supervision: Regina Ntabo, Cecilia Mbae

Validation: Sheillah Mundalo

Visualization: Sheillah Mundalo

Writing-original draft: Sheillah Mundalo

Writing-review& editing: Sheillah Mundalo, Cecilia Mbae, Regina Ntabo, Samuel Kariuki, Kelvin Kering, Rael Too, Diana Imoli, Brian Silantoi, Susan Kavai, Kevin Kariuki

Data availability

All data has been provided as part of the submitted article

Competing interest

The authors declare that they have no competing interests

Funding statement

This study received no specific funding

References

1. Faour-Klingbeil D, Todd ECD. Prevention and control of foodborne diseases in middle-east north african countries: Review of national control systems. Int J Environ Res Public Health. 2020;17(1):1–23.

2. Centre FM, Centre M. O[illegible]MIZING HUMAN HEALTH THROUGH. 2023;8(6):419–37.

3. Koudoum PL, Founou LL, Dimani BD, Mbossi A. Genomic Evidence for Extra-Intestinal Foodborne Disease due to Zoonotic Bacteria : A Public Health Risk. 2024;1–29.

4. TheLancet.

5. Abdullah T, Carr N, Lee C. The empowerment of street food vendors: a marginalised community within the hospitality industry. Tour Recreat Res [Internet]. 2023;48(6):885–98. Available from: https://doi.org/10.1080/02508281.2022.2076191

6. Mosisa D, Aboma M, Girma T, Shibru A. Determinants of diarrheal diseases among under five children in Jimma Geneti District, Oromia region, Ethiopia, 2020: a case-control study. BMC Pediatr [Internet]. 2021;21(1):1–13. Available from: https://doi.org/10.1186/s12887-021-03022-2

7. Samuel SK, Moses NM, Emily TJ. Prevalence of Enterobacteriaceae Isolated from Childhood Diarrhoea in Mukuru Slums, Nairobi- Kenya. J Adv Microbiol. 2019;17(3):1–9.

8. Mbuthia OW. Diarrheal correlates associated with enteric bacterial infections among children below five years in Murang'a County, Kenya. Pan Afr Med J. 2019;34:1–11.

9. Munhoz DD, Santos FF, Mitsunari T, Schüroff PA, Elias WP, Carvalho E, et al. Hybrid atypical enteropathogenic and extraintestinal escherichia coli (Aepec/expec) ba1250 strain: A draft genome. Pathogens. 2021;10(4).

10. Pakbin B, Brück WM, Allahyari S, Rossen JWA, Mahmoudi R. Antibiotic Resistance and Molecular Characterization of Cronobacter sakazakii Strains Isolated from Powdered Infant Formula Milk. Foods. 2022;11(8).

11. Amare A, Worku T, Ashagirie B, Adugna M, Getaneh A, Dagnew M. Bacteriological profile, antimicrobial susceptibility patterns of the isolates among street vended foods and hygienic practice of vendors in Gondar town, Northwest Ethiopia: A cross sectional study. BMC Microbiol. 2019;19(1):1–9.

12. Andersson DI, Balaban NQ, Baquero F, Courvalin P, Glaser P, Gophna U, et al. Antibiotic resistance: Turning evolutionary principles into clinical reality. FEMS Microbiol Rev. 2021;44(2):171–88.

13. OMS. WHO bacterial priority pathogens list, 2024 [Internet]. Bacterial pathogens of public health importance to guide research, development and strategies to prevent and control antimicrobial resistance. 2024. 72 p. Available from: https://www.who.int/publications/i/item/9789240093461

14. Vanderwall M, Strine MS. Antimicrobial Resistance. Yale J Biol Med. 2022;95(4):405–6.

15. Murray CJ, Ikuta KS, Sharara F, Swetschinski L, Robles Aguilar G, Gray A, et al. Global burden of bacterial antimicrobial resistance in 2019: a systematic analysis. Lancet. 2022;399(10325):629–55.

16. Michaelis C, Grohmann E. Horizontal Gene Transfer of Antibiotic Resistance Genes in Biofilms. Antibiotics. 2023;12(2).

17. Tufa TB, Regassa F, Amenu K, Stegeman JA, Hogeveen H. Livestock producers' knowledge, attitude, and behavior (KAB) regarding antimicrobial use in Ethiopia. Front Vet Sci. 2023;10:1–20.

18. Kasanga M, Shempela DM, Daka V, Mwikisa MJ, Sikalima J, Chanda D, et al. Antimicrobial resistance profiles of Escherichia coli isolated from clinical and environmental samples: findings and implications. JAC-Antimicrobial Resist [Internet]. 2024;6(2):1–10. Available from: https://doi.org/10.1093/jacamr/dlae061

19. Kiiru S, Maina J, Mwaniki JN, Songoro E, Kariuki S. Enteric bacterial agents associated with diarrhea and their antimicrobial sensitivity profiles in children under 5 years from mukuru informal settlement , Nairobi ,. 2024;1–10.

20. Corburn J, Agoe V, Ruiz M, Ortiz J, Patterson R, Wairitu J, et al. Mukuru | Nairobi, Kenya 2017 Situational Analysis: Mukuru Kwa Njenga, Kwa Reuben & Viwandani. 2017; Available from: http://sdinet.orghttp//akibamashinani.orghttp://urbanplanning.uonbi.ac.kehttp://www.strathmore.edu/en/

21. Amin N, Rahman M, Raj S, Ali S, Green J, Das S, et al. Quantitative assessment of fecal contamination in multiple environmental sample types in urban communities in Dhaka, Bangladesh using SaniPath microbial approach. PLoS One. 2019;14(12):1–21.

22. Dashti AA, Jadaon MM, Abdulsamad AM, Dashti HM. Heat treatment of bacteria: A simple method of DNA extraction for molecular techniques. Kuwait Med J.

2009;41(2):117–22.

23. Lorenz TC. Polymerase chain reaction: Basic protocol plus troubleshooting and optimization strategies. J Vis Exp. 2012;(63):1–15.

24. Gunzburg ST, Tornieporth NG, Riley LEEW. Identification of Enteropathogenic Escherichia coli by PCR-Based Detection of the Bundle-Forming Pilus Gene. 1995;33(5):1375–7.

25. Ihorimbere T, Kariuki S. Molecular Characteristics of Archived Isolates of Escherichia coli from the Gut of Healthy Food Animals and Environmental Sources in Selected Counties in Kenya. 2018;8(20):51–60.

26. Duong VT, Tu LTP, Tuyen HT, Nhi LTQ, Campbell JI, Van Minh P, et al. Correction: Novel multiplex real-time PCR assays reveal a high prevalence of diarrhoeagenic Escherichia coli pathotypes in healthy and diarrhoeal children in the south of Vietnam (BMC Microbiology, (2020), 20, 1, (192), 10.1186/s12866-020-01878-5). BMC Microbiol. 2022;22(1):1–12.

27. Compliance R. Clinical and Laboratory Standards Institute (CLSI) Regulatory Compliance CLIA and CLSI Standards Clarification. 2024;

28. Salamandane A, Malfeito-Ferreira M, Brito L. The Socioeconomic Factors of Street Food Vending in Developing Countries and Its Implications for Public Health: A Systematic Review. Foods. 2023;12(20).

29. Navab-Daneshmand T, Friedrich MND, Gächter M, Montealegre MC, Mlambo LS, Nhiwatiwa T, et al. Escherichia coli contamination across multiple environmental

compartments (soil, hands, drinking water, and handwashing water) in urban Harare: Correlations and risk factors. Am J Trop Med Hyg. 2018;98(3):803–13.

30. Hernández-Vásquez A, Visconti-Lopez FJ, Vargas-Fernández R. Escherichia coli Contamination of Water for Human Consumption and Its Associated Factors in Peru: A Cross-Sectional Study. Am J Trop Med Hyg. 2023;108(1):187–94.

31. Quality enhancement of potato chips through acrylamide mitigation and. 2024;8(December):48–56.

32. Fekadu Y, Kinde MZ, Dagnaw GG, Dessalegn B, Dejene H, Gessese AT. Knowledge, Attitude, and Practices on Food Safety among Food Handlers Working in Public Food Service Establishments in Lemi Kura Subcity, Addis Ababa, Ethiopia. Biomed Res Int. 2024;2024.

33. Bereda TW, Emerie YM, Reta MA, Asfaw HS. Microbiological Safety of Street Vended Foods in Jigjiga City, Eastern Ethiopia. Ethiop J Health Sci. 2016;26(2):161–70.

34. Nontongana N, Sibanda T, Ngwenya E, Okoh AI. Prevalence and antibiogram profiling of Escherichia coli pathotypes isolated from the kat river and the fort beaufort abstraction water. Int J Environ Res Public Health. 2014;11(8):8213–27.

35. Igwaran A, Iweriebor BC, Okoh AI. Molecular characterization and antimicrobial resistance pattern of Escherichia coli recovered from wastewater treatment plants in Eastern Cape South Africa. Int J Environ Res Public Health. 2018;15(6).

36. Bolukaoto JY, Singh A, Alfinete N, Barnard TG. Occurrence of hybrid diarrhoeagenic escherichia coli associated with multidrug resistance in environmental water,

johannesburg, south africa. Microorganisms. 2021;9(10).

37. Sukwa N, Bosomprah S, Somwe P, Muyoyeta M, Mwape K, Chibesa K, et al. The Incidence and Risk Factors for Enterotoxigenic E. coli Diarrheal Disease in Children under Three Years Old in Lusaka, Zambia. Microorganisms. 2024;12(4).

38. Some S, Mondal R, Mitra D, Jain D, Verma D, Das S. Microbial pollution of water with special reference to coliform bacteria and their nexus with environment. Energy Nexus [Internet]. 2021;1(May):100008. Available from: https://doi.org/10.1016/j.nexus.2021.100008

39. Carolina A, Santos DM, Santos FF, Silva RM. Diversity of Hybrid- and Hetero-Pathogenic Escherichia coli and Their Potential Implication in More Severe Diseases. 2020;10(July):1–11.

40. Bai X, Zhang J, Ambikan A, Jernberg C, Ehricht R, Scheutz F, et al. Molecular Characterization and Comparative Genomics of Clinical Hybrid Shiga Toxin-Producing and Enterotoxigenic Escherichia coli (STEC/ETEC) Strains in Sweden. Sci Rep [Internet]. 2019;9(1):1–9. Available from: http://dx.doi.org/10.1038/s41598-019-42122-z

41. CDC. Outbreak of Escherichia coli O104:H4 infections associated with sprout consumption - Europe and North America, May-July 2011. MMWR Morb Mortal Wkly Rep [Internet]. 2013;62(50):1029–31. Available from: http://www.ncbi.nlm.nih.gov/pubmed/24352067

42. Pakbin B, Amani Z, Allahyari S, Mousavi S, Mahmoudi R, Brück WM, et al. Genetic diversity and antibiotic resistance of Shigella spp. isolates from food products. Food Sci Nutr. 2021;9(11):6362–71.

43. Khalid S, Hussein S, Qurbani K, Hussein R, Fareeq A, Ali K, et al. Journal of Medicine , Surgery , and Public Health Antimicrobial resistance : Impacts , challenges , and future prospects. J Med Surgery, Public Heal [Internet]. 2024;2(January):100081. Available from: https://doi.org/10.1016/j.glmedi.2024.100081

44. Mukami SN. PHENOTYPIC AND MOLECULAR CHARACTERIZATION OF PATHOTYPES , AND ANTIMICROBIAL RESISTANCE PROFILES OF Escherichia coli FROM FOOD , WATER AND LIVESTOCK IN SELECTED INFORMAL SETTLEMENTS IN NAIROBI . Unversity of Nairobi. 2021;

45. Mshana SE, Sindato C, Matee MI, Mboera LEG. Antimicrobial Use and Resistance in Agriculture and Food Production Systems in Africa : A Systematic Review. 2021;

46. Shabani A, Rwiza MJ, Charles E, Miraji H, Linda N, Mzula A, et al. Environmental Chemistry and Ecotoxicology Antibiotic-resistant microbial populations in urban receiving waters and wastewaters from Tanzania. Environ Chem Ecotoxicol [Internet]. 2023;5(October 2022):1–8. Available from: https://doi.org/10.1016/j.enceco.2022.10.003

47. Islam MR, Rahman T, Alam J, Ashour HM. and Opportunities. 2023;1–22.

48. Davis MA, Baker KNK, Orfe LH, Shah DH, Besser TE, Call DR. Discovery of a Gene Conferring Multiple-Aminoglycoside Resistance in Escherichia coli □. 2010;54(6):2666–9.

49. Caneschi A, Bardhi A, Barbarossa A. The Use of Antibiotics and Antimicrobial Resistance in Veterinary Medicine , a Complex Phenomenon : A Narrative Review. 2023;

50. Food E, Authority S. The European Union summary report on antimicrobial resistance in

zoonotic and indicator bacteria from humans , animals and food in 2016. 2018;16(February).

51. Moyo P, Moyo E, Mangoya D, Mhango M. Prevention of antimicrobial resistance in sub-Saharan Africa : What has worked ? What still needs to be done ? J Infect Public Health [Internet]. 2023;16(4):632–9. Available from: https://doi.org/10.1016/j.jiph.2023.02.020

52. Vuuren V, Bogaard V Den. Pattern of antimicrobial usage in livestock animals in south-western Nigeria : The need for alternative plans. 2015;1–6.

53. Massele A, Rogers AM, Gabriel D, Mayanda A, Magoma S, Cook A, et al. A Narrative Review of Recent Antibiotic Prescribing Practices in Ambulatory Care in Tanzania : Findings and Implications. 2023;1–39.

54. Kunhikannan S, Thomas CJ, Franks AE, Mahadevaiah S, Kumar S, Petrovski S. Environmental hotspots for antibiotic resistance genes. 2021;(March):1–11.

55. Ribeiro LF, Augusto G, Rossi M, Sato RA, Pollo ADS, Cardozo MV, et al. Epidemiology , Virulence and Antimicrobial Resistance of Escherichia coli Isolated from Small Brazilian Farms Producers of Raw Milk Fresh Cheese. 2024;

56. Chaudhary MK, Jadhav I, Banjara MR. Molecular detection of plasmid mediated bla TEM , bla CTX − M , and bla SHV genes in Extended Spectrum β-Lactamase (ESBL) Escherichia coli from clinical samples. 2023;0:1–7.

Click here to access/download;Figure;Fig 1 Overral Prevelance of E.coli Pathotypes (1).tif

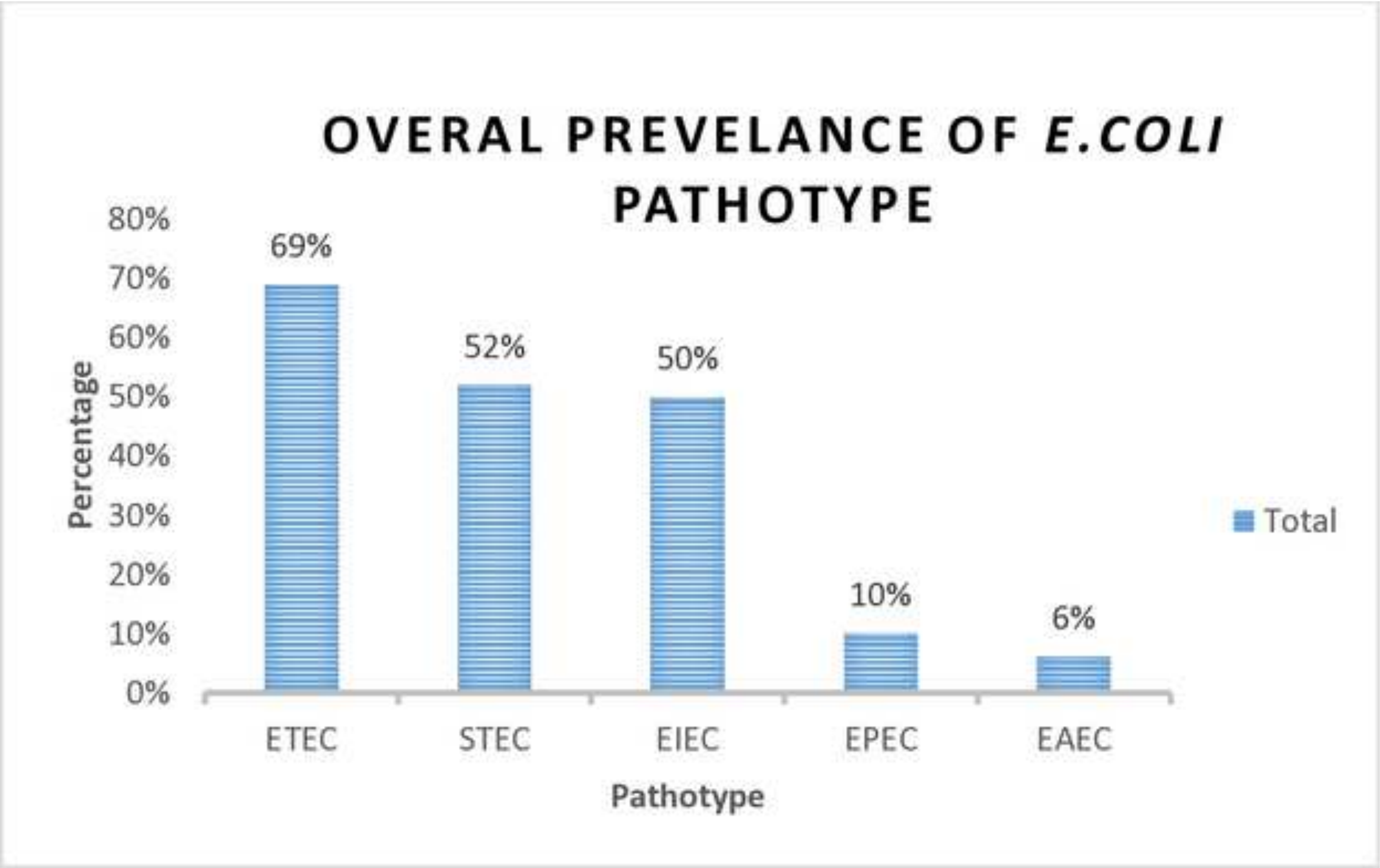

Key : EAEC- Enteroaggregative , STEC-Shiga-toxigenic, EIEC- Enteroinvasive , EPEC-Enteropathogenic and ETEC Enterotoxigenic .

 Click here to access/download;Figure;Fig 2. Prevelance of E.coli per sample.tif

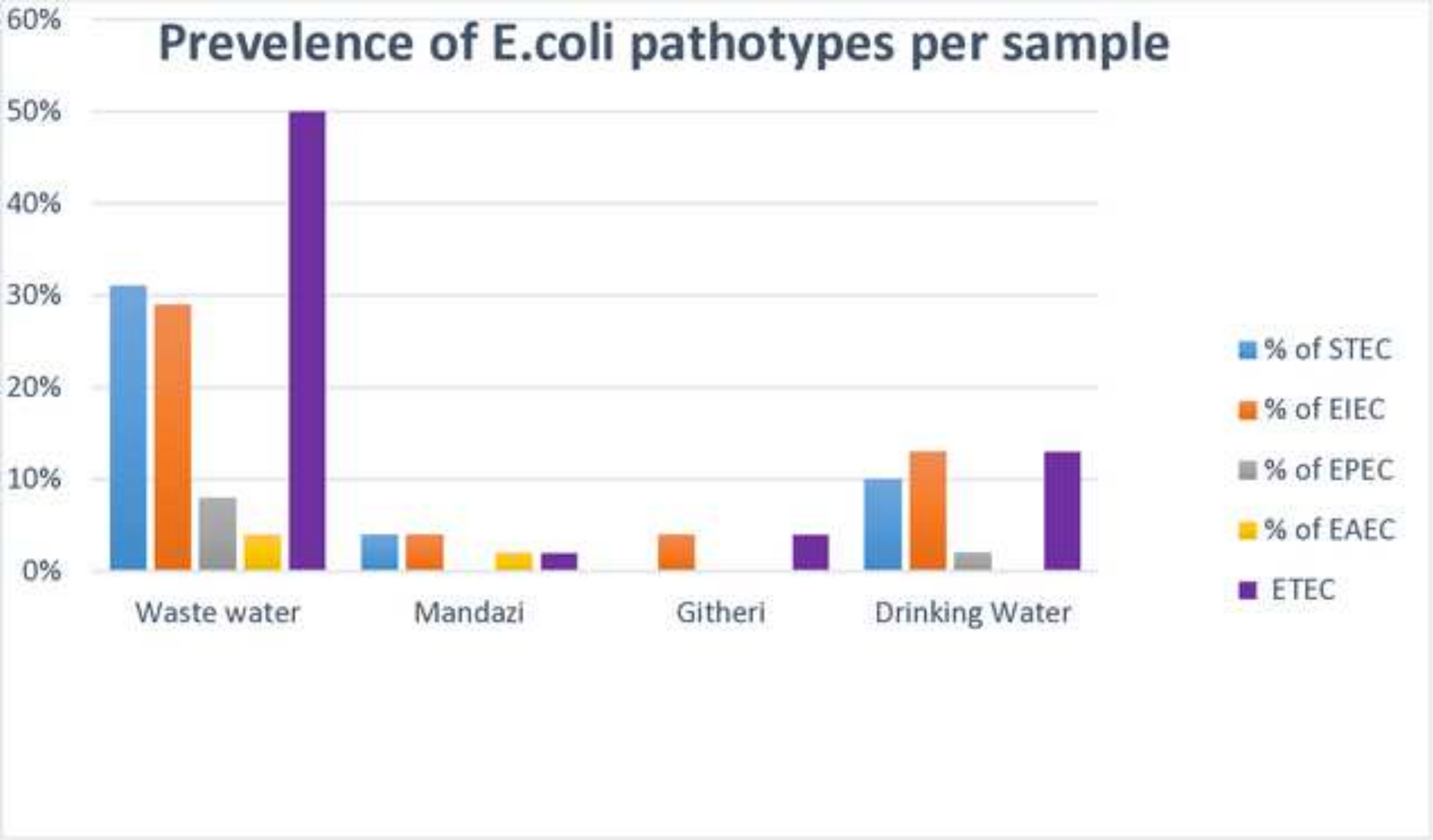

EAEC- Enteroaggregative , STEC-Shiga-toxigenic, EIEC- Enteroinvasive , EPEC-Enteropathogenic and ETEC Enterotoxigenic .

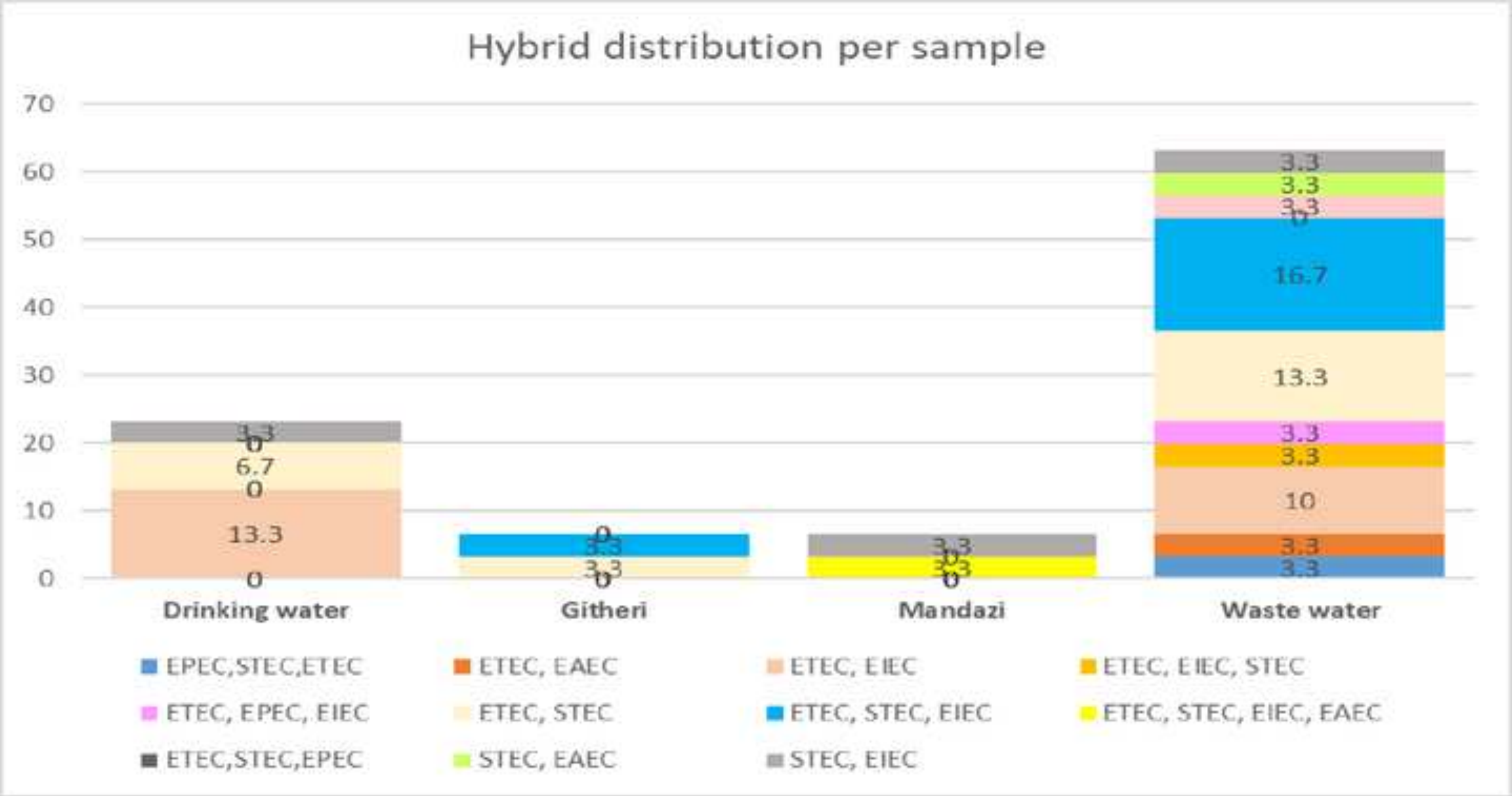

KEY: EAEC- Enteroaggregative , STEC-Shiga-toxigenic, EIEC- Enteroinvasive , EPEC- Enteropathogenic and ETEC Enterotoxigenic .

Figure Click here to access/download;Figure;Fig 4. Overall Hybrid pathotype distribution (1).tif

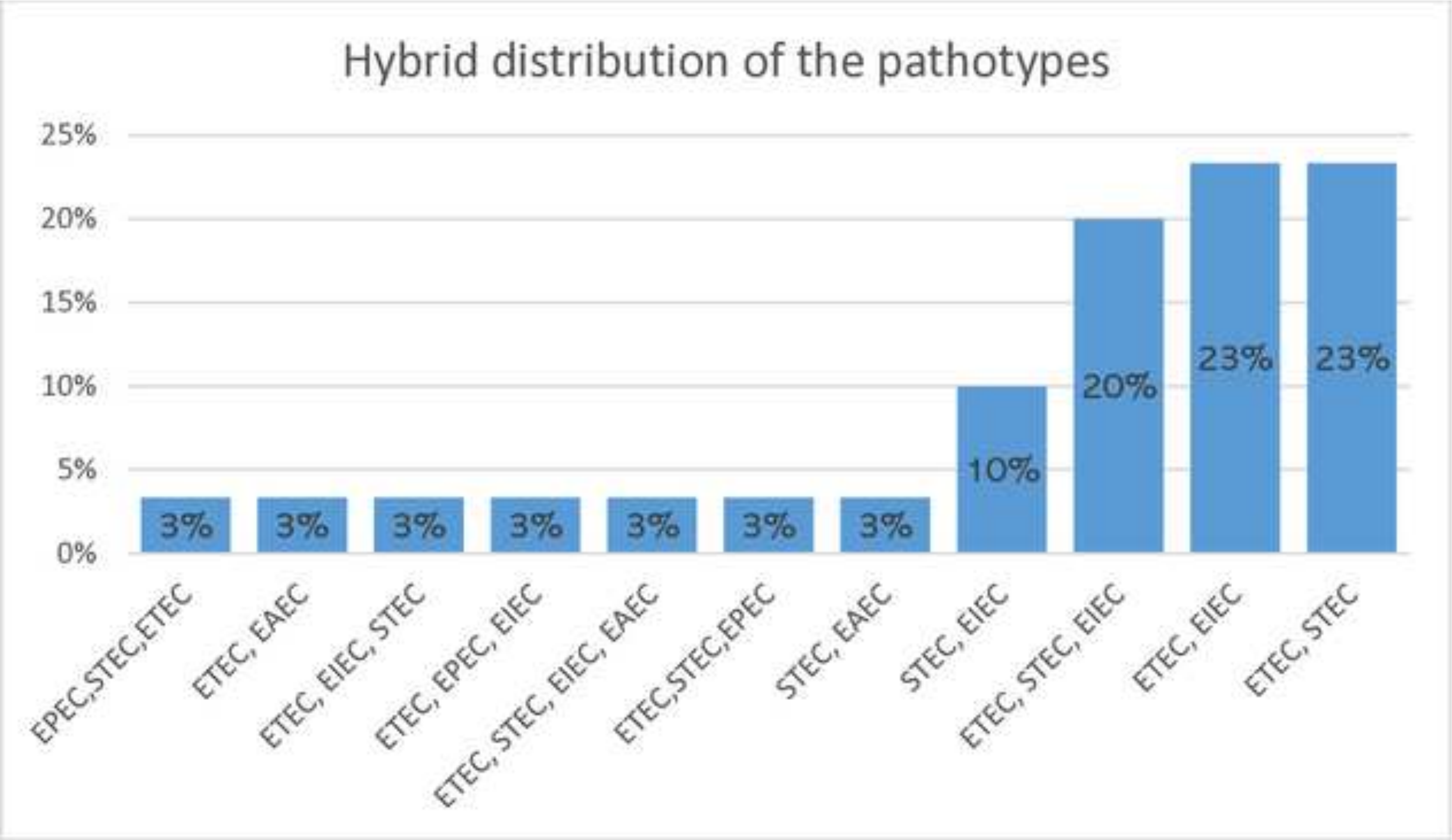

EAEC- Enteroaggregative , STEC-Shiga-toxigenic, EIEC- Enteroinvasive , EPEC-Enteropathogenic and ETEC Enterotoxigenic .

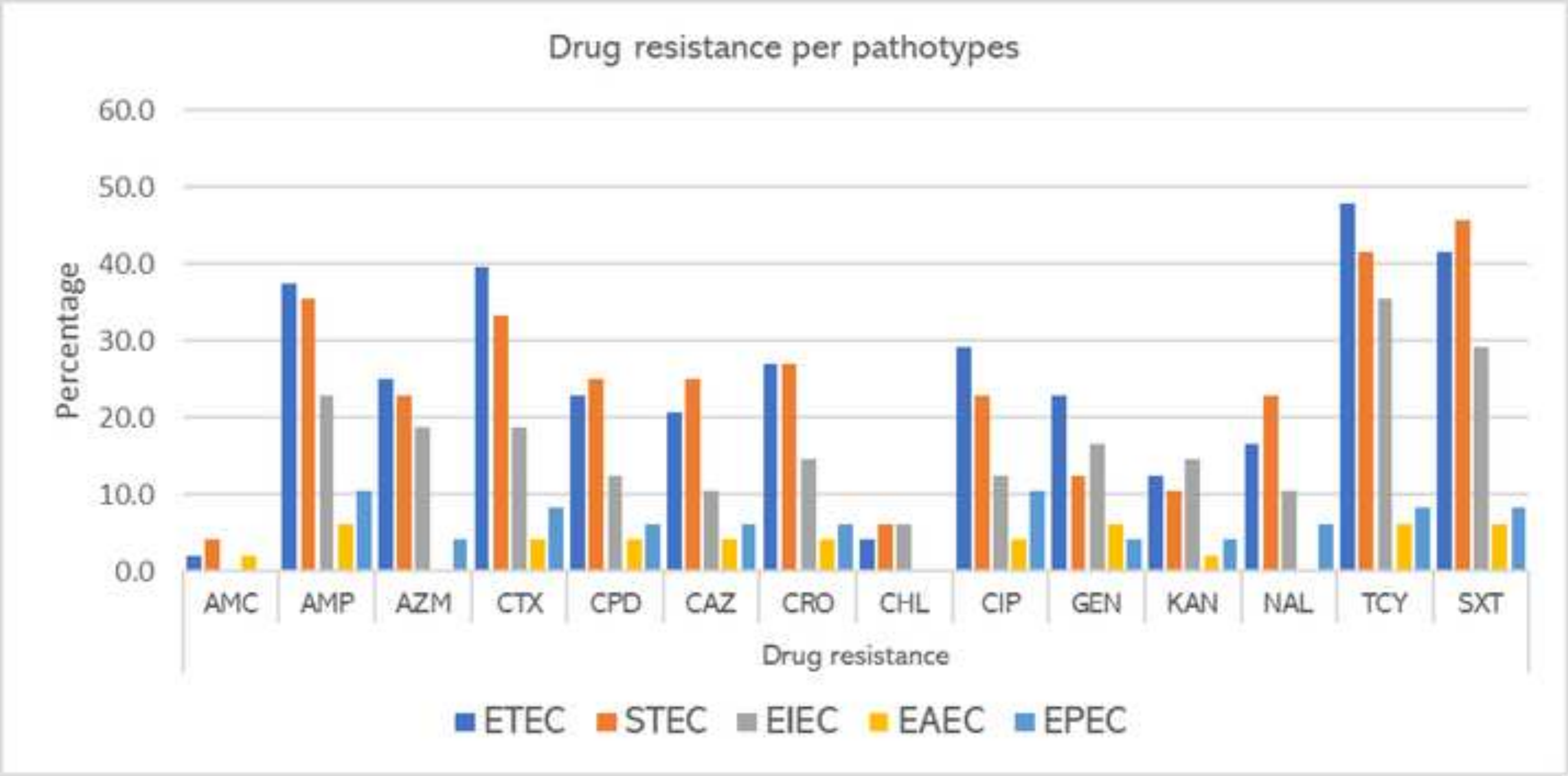

EAEC- Enteroaggregative , STEC-Shiga-toxigenic, EIEC- Enteroinvasive , EPEC-Enteropathogenic and ETEC Enterotoxigenic TCY -tetracycline, SXT -trimethoprim-sulfamethoxazole, AMP–ampicillin, CTX-cefotaxime, CIP –ciprofloxacin, AZM –azithromycin, CRO –ceftriaxone, CPD-cefpodoxime, GEN-gentamicin, CAZ –ceftazidime, NAL-nalidixic acid, KAN-kanamycin, CHL-chloramphenicol, AMC-amoxicillin-clavulanic acid

Figure

Click here to access/download;Figure;FIG.6.MDR E.COLI .tif

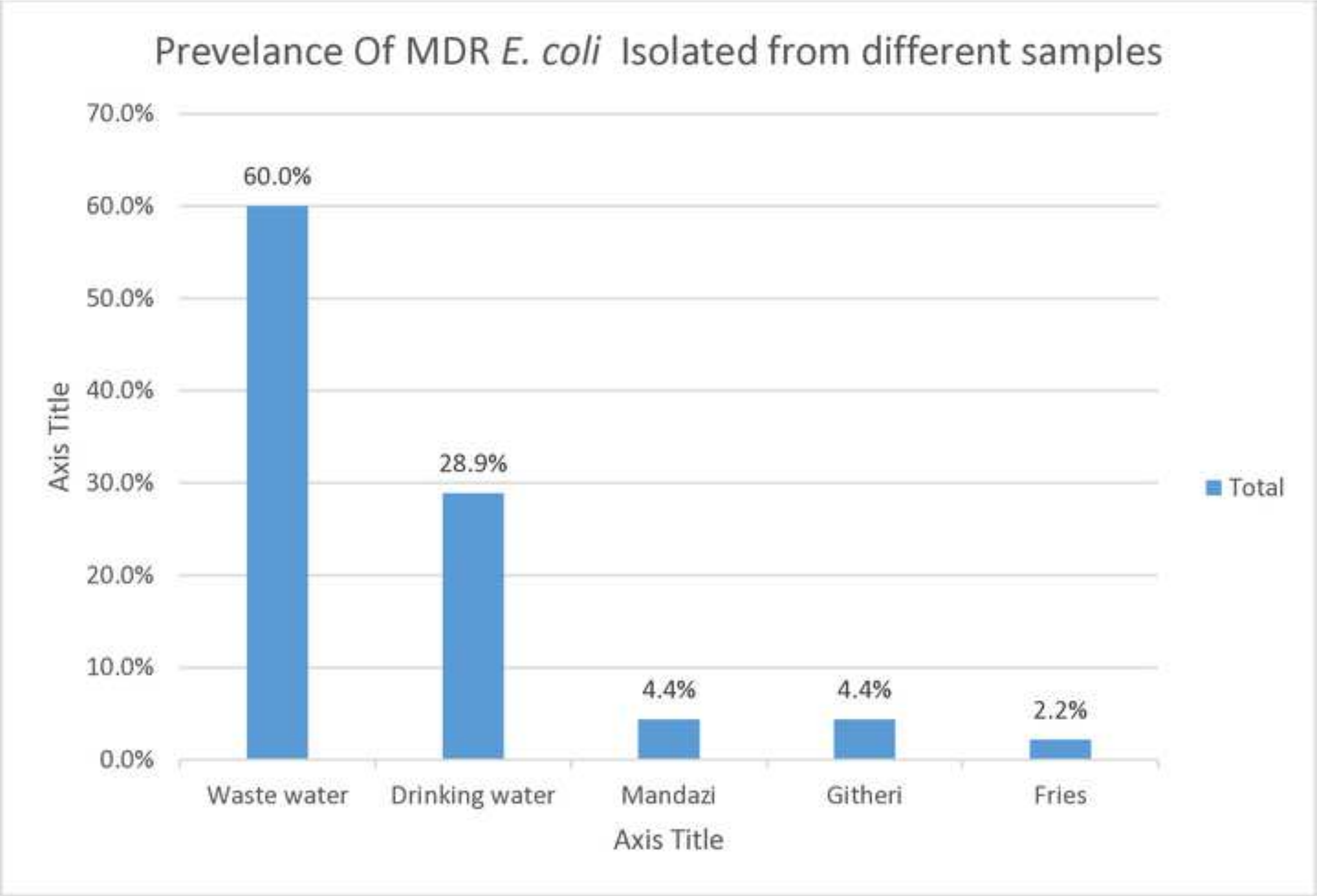

Click here to access/download;Figure;Fig.7 MDR Ecoli Per drug class.tif

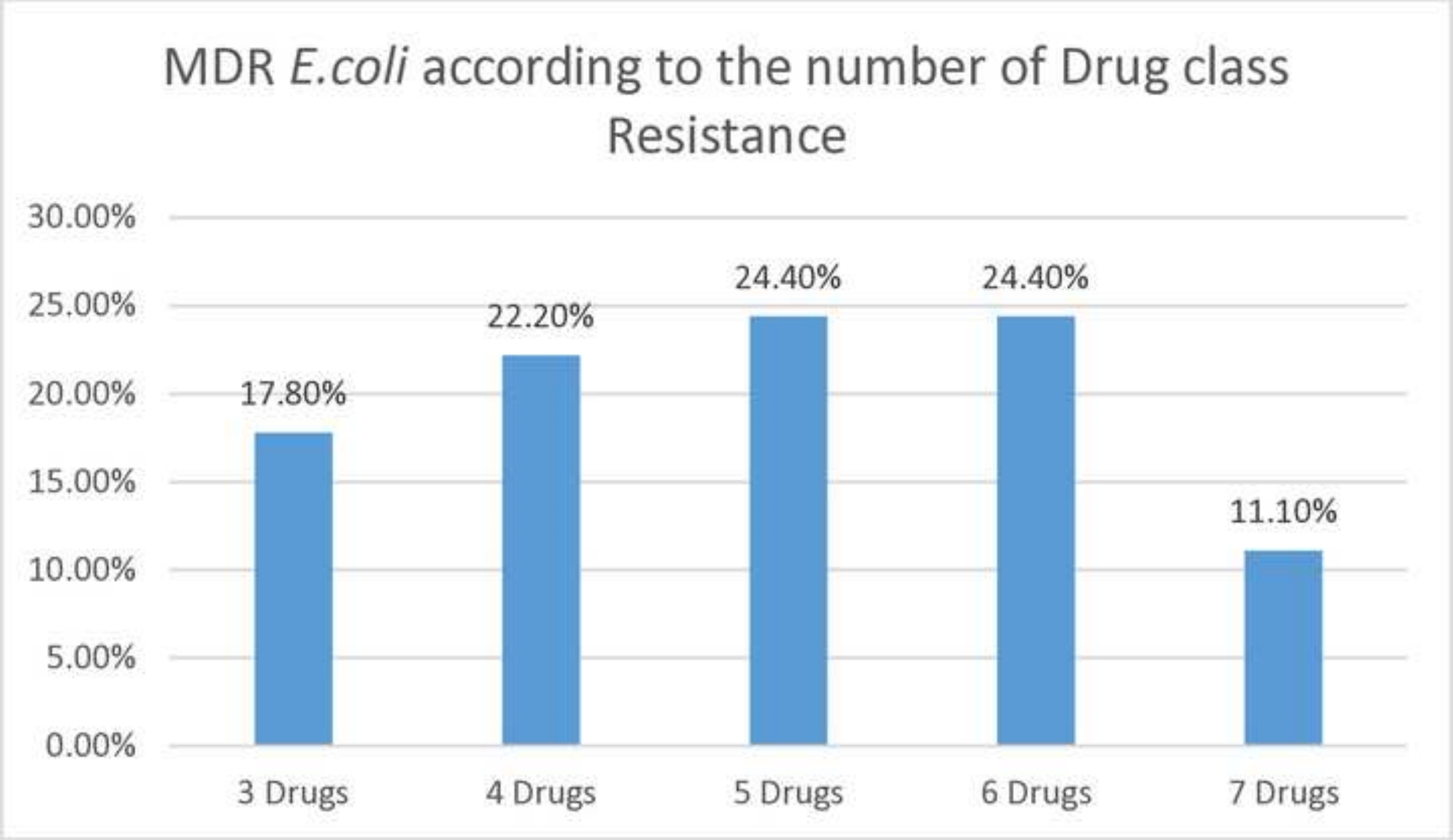

Click here to access/download
Supporting Information
Supporting Information.docx

Click here to access/download
Supporting Information
Multiplex Image (1) (2).tif

Click here to access/download
Supporting Information
S1.The Ingredients and Description of the Street Foods.docx

In Search of Better Health

KENYA MEDICAL RESEARCH INSTITUTE

24th March, 2025

Sheillah Mundalo
Kenya Medical Research Institute

TO
The Editor-in-Chief,
PLOS One.

Dear Sir/Madam,

We thank you for your decision on the manuscript **PONE-D-24-59070** titled **"Molecular Detection and Antibiotic Resistance of Diarrheagenic *Escherichia coli* from Street food and Water in Mukuru slums, Nairobi County".**

We also appreciate the reviewers' constructive and insightful comments. We have carefully considered the comments and revised the manuscript accordingly. We hope that with these improvements, the revised manuscript will satisfy the journal's requirements for publication. The following is a point-by-point list of our responses to the comments and changes made.

1. Is the manuscript technically sound, and do the data support the conclusions?

Reviewer #1: Yes

Reviewer #2: Partly

Reviewer #3: Yes

Response: We thank the reviewers for their thoughts on the manuscript as it has greatly improved the manuscript.

---

2. Has the statistical analysis been performed appropriately and rigorously?

Reviewer #1: Yes

Reviewer #2: I Don't Know

Reviewer #3: Yes.

Response: We appreciate the comments from reviewers. We believe that the revised data in the manuscript has been described methodologically and supports the conclusion.

---

3. Have the authors made all data underlying the findings in their manuscript fully available?

The PLOS Data policy requires authors to make all data underlying the findings described in their manuscript fully available without restriction, with rare exception (please refer to the Data Availability Statement in the manuscript PDF file). The data should be provided as part of the manuscript or its supporting information, or deposited to a public repository. For example, in addition to summary statistics, the data points behind means, medians and variance measures should be available. If there are restrictions on publicly sharing data—e.g. participant privacy or use of data from a third party—those must be specified.

Reviewer #1: Yes

Reviewer #2: Yes

Reviewer #3: Yes

Response: We wish to thank the reviewers again for taking time to review the manuscript. We believe that the statistical analysis done has provided in the manuscript and is rigorous.

---

4. Is the manuscript presented in an intelligible fashion and written in standard English?

PLOS ONE does not copyedit accepted manuscripts, so the language in submitted articles must be clear, correct, and unambiguous. Any typographical or grammatical errors should be corrected at revision, so please note any specific errors here.

Reviewer #1: Yes

Reviewer #2: No

Reviewer #3: Yes

Response: We appreciate the comments from reviewer #2. We have revised the manuscript accordingly and corrected the typographical and grammatical errors.

---

5. Review Comments to the Author

Please use the space provided to explain your answers to the questions above. You may also include additional comments for the author, including concerns about dual publication, research ethics, or publication ethics. (Please upload your review as an attachment if it exceeds 20,000 characters)

Reviewer #1:

While thanking you for your attention and inviting me to cooperate for intellectual participation in the field of reviewing and judging the manuscript entitled "Molecular Detection and Antibiotic Resistance of Diarrheagenic Escherichia coli from Street Food and Water in Mukuru slums, Nairobi County ", the suggested items to improve the scientific quality of the above article will be sent as follows:

1. Statistical analysis should be included in the abstract section of the article.

   Response: We appreciate the reviewer for this comment and agree with the suggestions. We have revised the abstract and included the statistical analysis in line 20-22. “We have included the statistical methods used in the revised manuscript.

2. The formula for determining sample size should be stated.
   Response: We thank the reviewer for highlighting this. We have revised and explained the Fischer formula that was used to determine the sample size in line 98-102.
3. The sources used in the methodology section must be included in the article.
   Response*:* We thank the reviewer for this comment and suggestion.The article referred to has been cited in line 93.
4. Old resources should be replaced.

The following articles should be used in the introduction and discussion section:

*Prevalence, phylogroups and antimicrobial susceptibility of escherichia coli isolates from food products

*Antibiotic Resistance and Molecular Characterization of Cronobacter sakazakii Strains Isolated from Powdered Infant Formula Milk

*PREVALENCE OF SALMONELLA STRAINS ISOLATED FROM INDUSTRIAL QUAIL EGGS AND LOCAL DUCK EGGS, IRAN

* Antibiotic susceptibility and genetic relatedness of Shigella species isolated from food and human stool samples in Qazvin, Iran

*Genetic diversity and antibiotic resistance of Shigella spp. isolates from food products

* Genotypic and antimicrobial resistance characterizations of Cronobacter sakazakii isolated from powdered milk infant formula: A comparison between domestic and imported products

Response*:* We thank the reviewer for this comment and suggestion.Old resources have been replaced and The suggested articles have been used to improve the manuscript

Reviewer #2:

Reviewer #2: Manuscript ID: PONE-D-24-59070

The manuscript titled "Molecular Detection and Antibiotic Resistance of Diarrheagenic Escherichia coli from Street food and Water in Mukuru slums, Nairobi County" focuses on the research, both phenotypic and genomic characterization of *E. coli* strains in wastewater, drinking water, and street food. The experimental approach adopted is not extremely innovative, but it allows the authors to collect numerous and important data.

However, I believe that the manuscript is not ready to be considered for publication because it has some important weaknesses described below.

Overall, the manuscript is not well written. The "Result" paragraph is extremely synthetic, so much so that it is not possible to understand why and how the results were calculated and expressed. The main weaknesses of the manuscript are Results and Discussion; furthermore, statistical analysis is sometimes mentioned in the text but there is no description of the analysis performed in "Materials and Method".

Abstract

1. Lines 21-23 The sum is not 100%, but 62.5. Please review the percentage calculations. Furthermore, and more seriously, there is no correspondence with the data reported in the text.
   Response: We appreciate the reviewer's comment. The calculations have been corrected, and they are now adding up to 100%.

2. The discrepancy between the data reported in the various points of the text is frequent. In fact, for example, in line 27 the authors say that tetracycline resistance is 70%, but in the text in line 194 the authors say it is 100%. Since it is not clear when in the text the authors refer to isolated E. coli or DEC, it is appropriate to underline this aspect.
   Response: We appreciate the reviewer for this insightful comment we have corrected the discrepancies and the results in the abstracts now match with results in 207-213. Phrases like isolated' have been added to sentences for clarity.

Introduction

3. There are some acronyms that should be explained (line 45 and line 72).

Response: We appreciate the reviewer for this comment and agree with the suggestions. We have revised the acronym 'LMIC' in line 45 as Low- and middle-income countries and 72 DEC as diarrheagenic *E. coli*

4. Lines 38-46. The numeration of bibliography in the text should be checked (the order is not respected).

Response: We appreciate the reviewer for this comment and agree with the suggestions. The numeration of bibliography has been corrected accordingly.

Materials and Methods

The materials and methods are concise but not sufficiently clear; several information seemed to be missing. More in details, the missing points and necessary corrections are reported below.

5. Table 1. In this table are listed more that genes mentioned in the text.

Response: We appreciate the reviewer for this comment . The table has been revised to reflect the genes mentioned in the text.

6. Lines 129-137. Review the text. Punctuation is missing and sometimes sentences don't make sense. Insert the weight of antibiotics.

Response: We appreciate the reviewer for this comment.The missing punctuations have been added and the weight of the antibiotics included.

7. Table 2: Insert in table R: and F:.

Response: We appreciate the reviewer for this comment.R and F have been inserted in the table.

Results

8. Table 3: check the percentage of Samples positive for E. coli pathotypes per Mandazi that is 2.6% and not 2.7%.

Response: We appreciate the reviewer for this comment.The mandazi percentage has been revised accordingly

9. Also, what kind of statistical analysis was performed? In M&M section the authors only talked about descriptive analysis

Response: We appreciate the reviewer for this comment. Inferential analysis, using chi-square statistics, was applied to assess associations.This has been added according in line 167.

10. Lines 179 – 180. How were these percentages calculated? It is not clear at all. Their sum is greater than 150% and there is no correspondence with figure 1. Furthermore, they do not correspond at all to the values reported in the abstract.

Response: We appreciate the reviewer for this comment. The percentages were calculated based on the total of 48 DEC positive samples. The values have been revised and they are now corresponding with those in the abstract and Figure 1.

11. Lines 179 - 183. Furthermore, the percentage of hybrid strains should be mentioned in this section, and it should be specified whether hybrid exclusion is taken into account in the calculation of pathotype percentages. In short, it is not clear whether these percentages are calculated on 48 (total DEC strains) or on 16 (no hybrid DEC strains).

Response: We appreciate the reviewer for this comment. The percentage of the hybrid strains as been added in the manuscript as advised. And it has been calculated based on the total of 30 Hybrid DEC-positive isolates.

12. Lines 194 – 198. This section needs to be rewritten because the subject and complement are reversed in the sentence structure (antibiotics are resistant to antibiotics, not vice versa). Additionally, this section needs to include data on resistance to all E. coli, DEC and others, because there is confusion between the resistance rates reported in the abstract and the text.

Response: We appreciate the reviewer for this comment.The section has been corrected accordingly as advised.

13. Lines 205 – 207. Please define what kind of statistics were performed to evaluate the significance of the results obtained.
Response: We appreciate the reviewer for this comment. Inferential analysis, using chi-square statistics, was applied to assess associations between sample type and the antibiotics. The same has also been added in the manuscript.
14. Lines 215 – 228. These paragraphs, like figures 4 and 5, should be completely revised since the concept of MDR is expressed in the resistance of the same bacterium to at least 3 pharmacological classes. From figure 5 for Fries and Mandazi it can be deduced that the strains are resistant to both SUL and TETRA, but the third pharmacological class involved is not intuitive and understandable. The text does not clarify anything about MDR strains, much less the figures that do nothing but represent the % of resistance to each of the pharmacological classes involved in the study.
Response: We appreciate the reviewer for this comment.The section has been corrected accordingly as advised.Fig 6 and 7 also shows the revised MDR aspect

15. Lines 232 – 233. In these lines, a further inconsistency is found between the data reported in the text and those in the table, in which 6 strains from wastewater and two from drinking water are positive for the tem gene.
Response: We appreciate the reviewer for this comment.The section has been corrected accordingly the data has been corrected and is now matching.

Discussion

16. Lines 261 – 262. How were these percentages calculated? Furthermore, they are still different from those reported in the abstract.

Response: We appreciate the reviewer for this comment.The section has been corrected accordingly the data has been corrected and is now matching.The percentages were calculated out of a total of 48 pathotypes.

17. Overall, the discussion is very repetitive. The weight of DEC and AMR on human health is repeatedly emphasized and reiterated. The concept is correct, but it is simply repeated. Some percentages referring to the results are reinserted, but with different values that once again create confusion in the reader.

Response: We appreciate the reviewer for this comment.The section has been corrected accordingly the data has been corrected and is now matching.

Figures

18. Fig. 1 The sum of the percentages is 99%. Furthermore, what does the 1.1% value reported on the orange bar of the wastewater samples (under 15.4) indicate?

Response: We appreciate the reviewer for this comment.The calculations have been revised and the sum is now 100%.The figure has also been revised.

19. Fig. 2 The sum of the percentages is 99.7%. However, if we are talking about hybrid why is ETEC (9.4%) reported in the wastewater bar?

Response: We appreciate the reviewer for this comment.The section has been corrected acordingly

20. Fig. 4 The figure can be avoided since it repeats data already listed in the results text. Also, together with figure 5, they do not represent MDR data, so they should be moved to other paragraphs.

Response: We appreciate the reviewer for this comment.The section has been corrected according and the figures also revised.

Reviewer #3: Dear Author

The summary of the article and the introduction are well-written; the methodology and the results presented in the tables indicate the scope of the work and are well articulated, with a good discussion as well.

There are just two points that need clarification: firstly, considering the number of genes studied,

21. why was a DNA extraction kit not used, and in the boiling method, the DNA purification is less efficient.

Response: We appreciate the reviewer for this comment.the project had no funding and thus boiling was the cheaper option.

22. Secondly, it would be beneficial to include an image of the multiplex gel, as it better illustrates the workflow.

Response: We appreciate the reviewer for this comment.The image has been included in the supplementary section.

Bacterial genera and species should be italicized in the text, tables, and references.

Response: We appreciate the reviewer for this comment.the bbacteria gens has been italicized.

Regards

7. PLOS authors have the option to publish the peer review history of their article (what does this mean?). If published, this will include your full peer review and any attached files.

Do you want your identity to be public for this peer review? If you choose “no”, your identity will remain anonymous but your review may still be made public.

For information about this choice, including consent withdrawal, please see our Privacy Policy.

Reviewer #1: No

Reviewer #2: No

Reviewer #3: No

Thank you for your consideration.

Kind regards,

Sheillah Mundalo

In Search of Better Health

KENYA MEDICAL RESEARCH INSTITUTE

24th March, 2025

Sheillah Mundalo
Kenya Medical Research Institute

TO
The Editor-in-Chief,
PLOS One.

Dear Sir/Madam,

We thank you for your decision on the manuscript **PONE-D-24-59070** titled **"Molecular Detection and Antibiotic Resistance of Diarrheagenic *Escherichia coli* from Street food and Water in Mukuru slums, Nairobi County".**

We also appreciate the reviewers' constructive and insightful comments. We have carefully considered the comments and revised the manuscript accordingly. We hope that with these improvements, the revised manuscript will satisfy the journal's requirements for publication. The following is a point-by-point list of our responses to the comments and changes made.

1. Is the manuscript technically sound, and do the data support the conclusions?

Reviewer #1: Yes

Reviewer #2: Partly

Reviewer #3: Yes

Response: We thank the reviewers for their thoughts on the manuscript as it has greatly improved the manuscript.

---

2. Has the statistical analysis been performed appropriately and rigorously?

Reviewer #1: Yes

Reviewer #2: I Don't Know

Reviewer #3: Yes.

Response: We appreciate the comments from reviewers. We believe that the revised data in the manuscript has been described methodologically and supports the conclusion.

---

3. Have the authors made all data underlying the findings in their manuscript fully available?

The PLOS Data policy requires authors to make all data underlying the findings described in their manuscript fully available without restriction, with rare exception (please refer to the Data Availability Statement in the manuscript PDF file). The data should be provided as part of the manuscript or its supporting information, or deposited to a public repository. For example, in addition to summary statistics, the data points behind means, medians and variance measures should be available. If there are restrictions on publicly sharing data—e.g. participant privacy or use of data from a third party—those must be specified.

Reviewer #1: Yes

Reviewer #2: Yes

Reviewer #3: Yes

Response: We wish to thank the reviewers again for taking time to review the manuscript. We believe that the statistical analysis done has provided in the manuscript and is rigorous.

---

4. Is the manuscript presented in an intelligible fashion and written in standard English?

PLOS ONE does not copyedit accepted manuscripts, so the language in submitted articles must be clear, correct, and unambiguous. Any typographical or grammatical errors should be corrected at revision, so please note any specific errors here.

Reviewer #1: Yes

Reviewer #2: No

Reviewer #3: Yes

Response: We appreciate the comments from reviewer #2. We have revised the manuscript accordingly and corrected the typographical and grammatical errors.

---

5. Review Comments to the Author

Please use the space provided to explain your answers to the questions above. You may also include additional comments for the author, including concerns about dual publication, research ethics, or publication ethics. (Please upload your review as an attachment if it exceeds 20,000 characters)

Reviewer #1:

While thanking you for your attention and inviting me to cooperate for intellectual participation in the field of reviewing and judging the manuscript entitled "Molecular Detection and Antibiotic Resistance of Diarrheagenic Escherichia coli from Street Food and Water in Mukuru slums, Nairobi County ", the suggested items to improve the scientific quality of the above article will be sent as follows:

1. Statistical analysis should be included in the abstract section of the article.

   Response: We appreciate the reviewer for this comment and agree with the suggestions. We have revised the abstract and included the statistical analysis in line 20-22. “We have included the statistical methods used in the revised manuscript.

2. The formula for determining sample size should be stated.
   Response: We thank the reviewer for highlighting this. We have revised and explained the Fischer formula that was used to determine the sample size in line 98-102.
3. The sources used in the methodology section must be included in the article.
   Response*:* We thank the reviewer for this comment and suggestion.The article referred to has been cited in line 93.
4. Old resources should be replaced.

The following articles should be used in the introduction and discussion section:

*Prevalence, phylogroups and antimicrobial susceptibility of escherichia coli isolates from food products

*Antibiotic Resistance and Molecular Characterization of Cronobacter sakazakii Strains Isolated from Powdered Infant Formula Milk

*PREVALENCE OF SALMONELLA STRAINS ISOLATED FROM INDUSTRIAL QUAIL EGGS AND LOCAL DUCK EGGS, IRAN

* Antibiotic susceptibility and genetic relatedness of Shigella species isolated from food and human stool samples in Qazvin, Iran

*Genetic diversity and antibiotic resistance of Shigella spp. isolates from food products

* Genotypic and antimicrobial resistance characterizations of Cronobacter sakazakii isolated from powdered milk infant formula: A comparison between domestic and imported products

Response*:* We thank the reviewer for this comment and suggestion.Old resources have been replaced and The suggested articles have been used to improve the manuscript

Reviewer #2:

Reviewer #2: Manuscript ID: PONE-D-24-59070

The manuscript titled "Molecular Detection and Antibiotic Resistance of Diarrheagenic Escherichia coli from Street food and Water in Mukuru slums, Nairobi County" focuses on the research, both phenotypic and genomic characterization of *E. coli* strains in wastewater, drinking water, and street food. The experimental approach adopted is not extremely innovative, but it allows the authors to collect numerous and important data.

However, I believe that the manuscript is not ready to be considered for publication because it has some important weaknesses described below.

Overall, the manuscript is not well written. The "Result" paragraph is extremely synthetic, so much so that it is not possible to understand why and how the results were calculated and expressed. The main weaknesses of the manuscript are Results and Discussion; furthermore, statistical analysis is sometimes mentioned in the text but there is no description of the analysis performed in "Materials and Method".

Abstract

1. Lines 21-23 The sum is not 100%, but 62.5. Please review the percentage calculations. Furthermore, and more seriously, there is no correspondence with the data reported in the text.
   Response: We appreciate the reviewer's comment. The calculations have been corrected, and they are now adding up to 100%.

2. The discrepancy between the data reported in the various points of the text is frequent. In fact, for example, in line 27 the authors say that tetracycline resistance is 70%, but in the text in line 194 the authors say it is 100%. Since it is not clear when in the text the authors refer to isolated E. coli or DEC, it is appropriate to underline this aspect.
   Response: We appreciate the reviewer for this insightful comment we have corrected the discrepancies and the results in the abstracts now match with results in 207-213. Phrases like isolated' have been added to sentences for clarity.

Introduction

3. There are some acronyms that should be explained (line 45 and line 72).

Response: We appreciate the reviewer for this comment and agree with the suggestions. We have revised the acronym 'LMIC' in line 45 as Low- and middle-income countries and 72 DEC as diarrheagenic *E. coli*

4. Lines 38-46. The numeration of bibliography in the text should be checked (the order is not respected).

Response: We appreciate the reviewer for this comment and agree with the suggestions. The numeration of bibliography has been corrected accordingly.

Materials and Methods

The materials and methods are concise but not sufficiently clear; several information seemed to be missing. More in details, the missing points and necessary corrections are reported below.

5. Table 1. In this table are listed more that genes mentioned in the text.

Response: We appreciate the reviewer for this comment . The table has been revised to reflect the genes mentioned in the text.

6. Lines 129-137. Review the text. Punctuation is missing and sometimes sentences don't make sense. Insert the weight of antibiotics.

Response: We appreciate the reviewer for this comment.The missing punctuations have been added and the weight of the antibiotics included.

7. Table 2: Insert in table R: and F:.

Response: We appreciate the reviewer for this comment.R and F have been inserted in the table.

Results

8. Table 3: check the percentage of Samples positive for E. coli pathotypes per Mandazi that is 2.6% and not 2.7%.

Response: We appreciate the reviewer for this comment.The mandazi percentage has been revised accordingly

9. Also, what kind of statistical analysis was performed? In M&M section the authors only talked about descriptive analysis

Response: We appreciate the reviewer for this comment. Inferential analysis, using chi-square statistics, was applied to assess associations.This has been added according in line 167.

10. Lines 179 – 180. How were these percentages calculated? It is not clear at all. Their sum is greater than 150% and there is no correspondence with figure 1. Furthermore, they do not correspond at all to the values reported in the abstract.

Response: We appreciate the reviewer for this comment. The percentages were calculated based on the total of 48 DEC positive samples. The values have been revised and they are now corresponding with those in the abstract and Figure 1.

11. Lines 179 - 183. Furthermore, the percentage of hybrid strains should be mentioned in this section, and it should be specified whether hybrid exclusion is taken into account in the calculation of pathotype percentages. In short, it is not clear whether these percentages are calculated on 48 (total DEC strains) or on 16 (no hybrid DEC strains).

Response: We appreciate the reviewer for this comment. The percentage of the hybrid strains as been added in the manuscript as advised. And it has been calculated based on the total of 30 Hybrid DEC-positive isolates.

12. Lines 194 – 198. This section needs to be rewritten because the subject and complement are reversed in the sentence structure (antibiotics are resistant to antibiotics, not vice versa). Additionally, this section needs to include data on resistance to all E. coli, DEC and others, because there is confusion between the resistance rates reported in the abstract and the text.

Response: We appreciate the reviewer for this comment.The section has been corrected accordingly as advised.

13. Lines 205 – 207. Please define what kind of statistics were performed to evaluate the significance of the results obtained.
    Response: We appreciate the reviewer for this comment. Inferential analysis, using chi-square statistics, was applied to assess associations between sample type and the antibiotics. The same has also been added in the manuscript.
14. Lines 215 – 228. These paragraphs, like figures 4 and 5, should be completely revised since the concept of MDR is expressed in the resistance of the same bacterium to at least 3 pharmacological classes. From figure 5 for Fries and Mandazi it can be deduced that the strains are resistant to both SUL and TETRA, but the third pharmacological class involved is not intuitive and understandable. The text does not clarify anything about MDR strains, much less the figures that do nothing but represent the % of resistance to each of the pharmacological classes involved in the study.
    Response: We appreciate the reviewer for this comment.The section has been corrected accordingly as advised.Fig 6 and 7 also shows the revised MDR aspect

15. Lines 232 – 233. In these lines, a further inconsistency is found between the data reported in the text and those in the table, in which 6 strains from wastewater and two from drinking water are positive for the tem gene.
    Response: We appreciate the reviewer for this comment.The section has been corrected accordingly the data has been corrected and is now matching.

Discussion

16. Lines 261 – 262. How were these percentages calculated? Furthermore, they are still different from those reported in the abstract.

Response: We appreciate the reviewer for this comment.The section has been corrected accordingly the data has been corrected and is now matching.The percentages were calculated out of a total of 48 pathotypes.

17. Overall, the discussion is very repetitive. The weight of DEC and AMR on human health is repeatedly emphasized and reiterated. The concept is correct, but it is simply repeated. Some percentages referring to the results are reinserted, but with different values that once again create confusion in the reader.

Response: We appreciate the reviewer for this comment.The section has been corrected accordingly the data has been corrected and is now matching.

Figures

18. Fig. 1 The sum of the percentages is 99%. Furthermore, what does the 1.1% value reported on the orange bar of the wastewater samples (under 15.4) indicate?

Response: We appreciate the reviewer for this comment.The calculations have been revised and the sum is now 100%.The figure has also been revised.

19. Fig. 2 The sum of the percentages is 99.7%. However, if we are talking about hybrid why is ETEC (9.4%) reported in the wastewater bar?

Response: We appreciate the reviewer for this comment.The section has been corrected acordingly

20. Fig. 4 The figure can be avoided since it repeats data already listed in the results text. Also, together with figure 5, they do not represent MDR data, so they should be moved to other paragraphs.

Response: We appreciate the reviewer for this comment.The section has been corrected according and the figures also revised.

Reviewer #3: Dear Author

The summary of the article and the introduction are well-written; the methodology and the results presented in the tables indicate the scope of the work and are well articulated, with a good discussion as well.

There are just two points that need clarification: firstly, considering the number of genes studied,

21. why was a DNA extraction kit not used, and in the boiling method, the DNA purification is less efficient.

Response: We appreciate the reviewer for this comment.the project had no funding and thus boiling was the cheaper option.

22. Secondly, it would be beneficial to include an image of the multiplex gel, as it better illustrates the workflow.

Response: We appreciate the reviewer for this comment.The image has been included in the supplementary section.

Bacterial genera and species should be italicized in the text, tables, and references.

Response: We appreciate the reviewer for this comment.the bbacteria gens has been italicized.

Regards

7. PLOS authors have the option to publish the peer review history of their article (what does this mean?). If published, this will include your full peer review and any attached files.

Do you want your identity to be public for this peer review? If you choose “no”, your identity will remain anonymous but your review may still be made public.

For information about this choice, including consent withdrawal, please see our Privacy Policy.

Reviewer #1: No

Reviewer #2: No

Reviewer #3: No

Thank you for your consideration.

Kind regards,

Sheillah Mundalo
