# Supplementary material for: Molecular detection and antibiotic resistance of diarrheagenic Escherichia coli from street food and water in mukuru slums, Nairobi County
Source: PLoS One. 2026 Jan 28;21(1):e0340081. doi: 10.1371/journal.pone.0340081 (PMC12851472; doi:10.1371/journal.pone.0340081)
Supplement: Attachment — Submitted filename: PONE-D-24-59070.docx [file pone.0340081.s002.docx]

**Dear**

Although the article does not report a new method or any specific innovation and is primarily focused on the identification of bacteria and antibiotic resistance, this subject is more local or regional and can vary in different areas depending on the hygiene of that region.

However, given the importance of public health, the topic is undeniable and can be chosen for publication. The summary of the article and the introduction are well-written; the methodology and the results presented in the tables indicate the scope of the work and are well articulated, with a good discussion as well.

There are just two points that need clarification: firstly, considering the number of genes studied, why was a DNA extraction kit not used, and in the boiling method, the DNA purification is less efficient. Secondly, it would be beneficial to include an image of the multiplex gel, as it better illustrates the workflow.
