# Supplementary material for: Molecular detection and antibiotic resistance of diarrheagenic Escherichia coli from street food and water in mukuru slums, Nairobi County
Source: PLoS One. 2026 Jan 28;21(1):e0340081. doi: 10.1371/journal.pone.0340081 (PMC12851472; doi:10.1371/journal.pone.0340081)
Supplement: Attachment — Submitted filename: Response to reviewer.docx [file pone.0340081.s006.docx]

**KENYA MEDICAL RESEARCH INSTITUTE**

24^th^ March, 2025

Sheillah Mundalo

Kenya Medical Research Institute

TO

The Editor-in-Chief,

PLOS One.

Dear Sir/Madam,

We thank you for your decision on the manuscript **PONE-D-24-59070** titled **“Molecular Detection and Antibiotic Resistance of Diarrheagenic *Escherichia coli* from Street food and Water in Mukuru slums, Nairobi County”.**

We also appreciate the reviewers’ constructive and insightful comments. We have carefully considered the comments and revised the manuscript accordingly. We hope that with these improvements, the revised manuscript will satisfy the journal’s requirements for publication. The following is a point-by-point list of our responses to the comments and changes made.

1. Is the manuscript technically sound, and do the data support the conclusions?

Reviewer #1: Yes

Reviewer #2: Partly

Reviewer #3: Yes

**Response:** We thank the reviewers for their thoughts on the manuscript as it has greatly improved the manuscript.

2. Has the statistical analysis been performed appropriately and rigorously?

Reviewer #1: Yes

Reviewer #2: I Don't Know

Reviewer #3: Yes.

**Response:** We appreciate the comments from reviewers. We believe that the revised data in the manuscript has been described methodologically and supports the conclusion.

3. Have the authors made all data underlying the findings in their manuscript fully available?

The PLOS Data policy requires authors to make all data underlying the findings described in their manuscript fully available without restriction, with rare exception (please refer to the Data Availability Statement in the manuscript PDF file). The data should be provided as part of the manuscript or its supporting information, or deposited to a public repository. For example, in addition to summary statistics, the data points behind means, medians and variance measures should be available. If there are restrictions on publicly sharing data—e.g. participant privacy or use of data from a third party—those must be specified.

Reviewer #1: Yes

Reviewer #2: Yes

Reviewer #3: Yes

**Response**: We wish to thank the reviewers again for taking time to review the manuscript. We believe that the statistical analysis done has provided in the manuscript and is rigorous.

4. Is the manuscript presented in an intelligible fashion and written in standard English?

PLOS ONE does not copyedit accepted manuscripts, so the language in submitted articles must be clear, correct, and unambiguous. Any typographical or grammatical errors should be corrected at revision, so please note any specific errors here.

Reviewer #1: Yes

Reviewer #2: No

Reviewer #3: Yes

**Response:** We appreciate the comments from reviewer #2. We have revised the manuscript accordingly and corrected the typographical and grammatical errors.

5. Review Comments to the Author

Please use the space provided to explain your answers to the questions above. You may also include additional comments for the author, including concerns about dual publication, research ethics, or publication ethics. (Please upload your review as an attachment if it exceeds 20,000 characters)

**Reviewer #1**:

While thanking you for your attention and inviting me to cooperate for intellectual participation in the field of reviewing and judging the manuscript entitled "Molecular Detection and Antibiotic Resistance of Diarrheagenic Escherichia coli from Street Food and Water in Mukuru slums, Nairobi County ", the suggested items to improve the scientific quality of the above article will be sent as follows:

1. Statistical analysis should be included in the abstract section of the article.

**Response**: We appreciate the reviewer for this comment and agree with the suggestions. We have revised the abstract and included the statistical analysis in line 20-22. “We have included the statistical methods used in the revised manuscript.

2. The formula for determining sample size should be stated.

**Response:** We thank the reviewer for highlighting this. We have revised and explained the Fischer formula that was used to determine the sample size in line 98-102.

3. The sources used in the methodology section must be included in the article.

**Response***:* We thank the reviewer for this comment and suggestion.The article referred to has been cited in line 93.

4. Old resources should be replaced.

The following articles should be used in the introduction and discussion section:

*Prevalence, phylogroups and antimicrobial susceptibility of escherichia coli isolates from food products

*Antibiotic Resistance and Molecular Characterization of Cronobacter sakazakii Strains Isolated from Powdered Infant Formula Milk

*PREVALENCE OF SALMONELLA STRAINS ISOLATED FROM INDUSTRIAL QUAIL EGGS AND LOCAL DUCK EGGS, IRAN

* Antibiotic susceptibility and genetic relatedness of Shigella species isolated from food and human stool samples in Qazvin, Iran

*Genetic diversity and antibiotic resistance of Shigella spp. isolates from food products

* Genotypic and antimicrobial resistance characterizations of Cronobacter sakazakii isolated from powdered milk infant formula: A comparison between domestic and imported products

**Response***:* We thank the reviewer for this comment and suggestion.Old resources have been replaced and The suggested articles have been used to improve the manuscript

**Reviewer #2:**

Reviewer #2: Manuscript ID: PONE-D-24-59070

The manuscript titled "Molecular Detection and Antibiotic Resistance of Diarrheagenic Escherichia coli from Street food and Water in Mukuru slums, Nairobi County" focuses on the research, both phenotypic and genomic characterization of *E. coli* strains in wastewater, drinking water, and street food. The experimental approach adopted is not extremely innovative, but it allows the authors to collect numerous and important data.

However, I believe that the manuscript is not ready to be considered for publication because it has some important weaknesses described below.

Overall, the manuscript is not well written. The "Result" paragraph is extremely synthetic, so much so that it is not possible to understand why and how the results were calculated and expressed. The main weaknesses of the manuscript are Results and Discussion; furthermore, statistical analysis is sometimes mentioned in the text but there is no description of the analysis performed in "Materials and Method".

Abstract

1. Lines 21-23 The sum is not 100%, but 62.5. Please review the percentage calculations. Furthermore, and more seriously, there is no correspondence with the data reported in the text.

**Response**: We appreciate the reviewer's comment. The calculations have been corrected, and they are now adding up to 100%.

2. The discrepancy between the data reported in the various points of the text is frequent. In fact, for example, in line 27 the authors say that tetracycline resistance is 70%, but in the text in line 194 the authors say it is 100%. Since it is not clear when in the text the authors refer to isolated E. coli or DEC, it is appropriate to underline this aspect.

**Response**: We appreciate the reviewer for this insightful comment we have corrected the discrepancies and the results in the abstracts now match with results in 207-213. Phrases like isolated’ have been added to sentences for clarity.

Introduction

3. There are some acronyms that should be explained (line 45 and line 72).

**Response**: We appreciate the reviewer for this comment and agree with the suggestions. We have revised the acronym ‘LMIC’ in line 45 as Low- and middle-income countries and 72 DEC as diarrheagenic *E. coli*

4. Lines 38-46. The numeration of bibliography in the text should be checked (the order is not respected).

**Response**: We appreciate the reviewer for this comment and agree with the suggestions. The numeration of bibliography has been corrected accordingly.

Materials and Methods

The materials and methods are concise but not sufficiently clear; several information seemed to be missing. More in details, the missing points and necessary corrections are reported below.

5. Table 1. In this table are listed more that genes mentioned in the text.

**Response**: We appreciate the reviewer for this comment . The table has been revised to reflect the genes mentioned in the text.

6. Lines 129-137. Review the text. Punctuation is missing and sometimes sentences don't make sense. Insert the weight of antibiotics.

**Response**: We appreciate the reviewer for this comment.The missing punctuations have been added and the weight of the antibiotics included.

7. Table 2: Insert in table R: and F:.

**Response**: We appreciate the reviewer for this comment.R and F have been inserted in the table.

Results

8. Table 3: check the percentage of Samples positive for E. coli pathotypes per Mandazi that is 2.6% and not 2.7%.

**Response**: We appreciate the reviewer for this comment.The mandazi percentage has been revised accordingly

9. Also, what kind of statistical analysis was performed? In M&M section the authors only talked about descriptive analysis

**Response**: We appreciate the reviewer for this comment. Inferential analysis, using chi-square statistics, was applied to assess associations.This has been added according in line 167.

10. Lines 179 – 180. How were these percentages calculated? It is not clear at all. Their sum is greater than 150% and there is no correspondence with figure 1. Furthermore, they do not correspond at all to the values reported in the abstract.

**Respons**e: We appreciate the reviewer for this comment. The percentages were calculated based on the total of 48 DEC positive samples. The values have been revised and they are now corresponding with those in the abstract and Figure 1.

11. Lines 179 - 183. Furthermore, the percentage of hybrid strains should be mentioned in this section, and it should be specified whether hybrid exclusion is taken into account in the calculation of pathotype percentages. In short, it is not clear whether these percentages are calculated on 48 (total DEC strains) or on 16 (no hybrid DEC strains).

**Respons**e: We appreciate the reviewer for this comment. The percentage of the hybrid strains as been added in the manuscript as advised. And it has been calculated based on the total of 30 Hybrid DEC-positive isolates.

12. Lines 194 – 198. This section needs to be rewritten because the subject and complement are reversed in the sentence structure (antibiotics are resistant to antibiotics, not vice versa). Additionally, this section needs to include data on resistance to all E. coli, DEC and others, because there is confusion between the resistance rates reported in the abstract and the text.

**Respons**e: We appreciate the reviewer for this comment.The section has been corrected accordingly as advised.

13. Lines 205 – 207. Please define what kind of statistics were performed to evaluate the significance of the results obtained.

**Respons**e: We appreciate the reviewer for this comment. Inferential analysis, using chi-square statistics, was applied to assess associations between sample type and the antibiotics. The same has also been added in the manuscript.

14. Lines 215 – 228. These paragraphs, like figures 4 and 5, should be completely revised since the concept of MDR is expressed in the resistance of the same bacterium to at least 3 pharmacological classes. From figure 5 for Fries and Mandazi it can be deduced that the strains are resistant to both SUL and TETRA, but the third pharmacological class involved is not intuitive and understandable. The text does not clarify anything about MDR strains, much less the figures that do nothing but represent the % of resistance to each of the pharmacological classes involved in the study.

**Respons**e: We appreciate the reviewer for this comment.The section has been corrected accordingly as advised.Fig 6 and 7 also shows the revised MDR aspect

15. Lines 232 – 233. In these lines, a further inconsistency is found between the data reported in the text and those in the table, in which 6 strains from wastewater and two from drinking water are positive for the tem gene.

**Respons**e: We appreciate the reviewer for this comment.The section has been corrected accordingly the data has been corrected and is now matching.

Discussion

16. Lines 261 – 262. How were these percentages calculated? Furthermore, they are still different from those reported in the abstract.

**Respons**e: We appreciate the reviewer for this comment.The section has been corrected accordingly the data has been corrected and is now matching.The percentages were calculated out of a total of 48 pathotypes.

17. Overall, the discussion is very repetitive. The weight of DEC and AMR on human health is repeatedly emphasized and reiterated. The concept is correct, but it is simply repeated. Some percentages referring to the results are reinserted, but with different values that once again create confusion in the reader.

**Respons**e: We appreciate the reviewer for this comment.The section has been corrected accordingly the data has been corrected and is now matching.

Figures

18. Fig. 1 The sum of the percentages is 99%. Furthermore, what does the 1.1% value reported on the orange bar of the wastewater samples (under 15.4) indicate?

**Respons**e: We appreciate the reviewer for this comment.The calculations have been revised and the sum is now 100%.The figure has also been revised.

19. Fig. 2 The sum of the percentages is 99.7%. However, if we are talking about hybrid why is ETEC (9.4%) reported in the wastewater bar?

**Respons**e: We appreciate the reviewer for this comment.The section has been corrected acordingly

20. Fig. 4 The figure can be avoided since it repeats data already listed in the results text. Also, together with figure 5, they do not represent MDR data, so they should be moved to other paragraphs.

**Response:** We appreciate the reviewer for this comment.The section has been corrected according and the figures also revised.

Reviewer #3: Dear Author

The summary of the article and the introduction are well-written; the methodology and the results presented in the tables indicate the scope of the work and are well articulated, with a good discussion as well.

There are just two points that need clarification: firstly, considering the number of genes studied,

21. why was a DNA extraction kit not used, and in the boiling method, the DNA purification is less efficient.

**Response:** We appreciate the reviewer for this comment.the project had no funding and thus boiling was the cheaper option.

22. Secondly, it would be beneficial to include an image of the multiplex gel, as it better illustrates the workflow.

**Response: We** appreciate the reviewer for this comment.The image has been included in the supplementary section.

Bacterial genera and species should be italicized in the text, tables, and references.

**Response:** We appreciate the reviewer for this comment.the bbacteria gens has been italicized.

Regards

7. PLOS authors have the option to publish the peer review history of their article ([what does this mean?](https://journals.plos.org/globalpublichealth/s/editorial-and-peer-review-process#loc-peer-review-history)). If published, this will include your full peer review and any attached files.


**Do you want your identity to be public for this peer review?** If you choose “no”, your identity will remain anonymous but your review may still be made public.


For information about this choice, including consent withdrawal, please see our [Privacy Policy](https://www.plos.org/privacy-policy).

Reviewer #1: No

Reviewer #2: No

Reviewer #3: No

Thank you for your consideration.

Kind regards,

Sheillah Mundalo
