# Supplementary material for: Molecular detection and antibiotic resistance of diarrheagenic Escherichia coli from street food and water in mukuru slums, Nairobi County
Source: PLoS One. 2026 Jan 28;21(1):e0340081. doi: 10.1371/journal.pone.0340081 (PMC12851472; doi:10.1371/journal.pone.0340081)
Supplement: Attachment — Submitted filename: Plos One- Mundalo et al- Review Comments .docx [file pone.0340081.s005.docx]

**Journal Article Review- Revision 2**

**Molecular Detection and Antibiotic Resistance of Diarrheagenic Escherichia coli from Street food and Water in Mukuru slums, Nairobi County**

The manuscript presents a cross-sectional study assessing the occurrence, pathotyping, and antimicrobial resistance (AMR) profiles of diarrheagenic *Escherichia coli* (DEC) in street food and water samples collected from the Mukuru informal settlements in Nairobi, Kenya. The topic is timely and important, especially considering the growing public health threat posed by antimicrobial resistance and fecal contamination in low-resource urban settings.

Although I was not involved in the initial round of peer review for this manuscript, it appears that the authors have adequately addressed the concerns and suggestions raised during the initial review process. The revisions are thoughtful, and the manuscript has been improved as a result. However, I’ve the following suggestions:

1. The figure captions and corresponding explanations are not adequate. Provide specific details corresponding to each figure.

2. Provide appropriate Axis titles for figures 2,3,4,6 and 7.

3. Streamline content in the Results and Discussion section to avoid redundancy and focus on key insights specific to this setting.
