# Supplementary material for: Molecular detection and antibiotic resistance of diarrheagenic Escherichia coli from street food and water in mukuru slums, Nairobi County
Source: PLoS One. 2026 Jan 28;21(1):e0340081. doi: 10.1371/journal.pone.0340081 (PMC12851472; doi:10.1371/journal.pone.0340081)
Supplement: Attachment — Submitted filename: RESPONSE TO SECOND REVIEWERS.docx [file pone.0340081.s007.docx]

**KENYA MEDICAL RESEARCH INSTITUTE**

27^th^ July, 2025

Sheillah Mundalo

Kenya Medical Research Institute

TO

The Editor-in-Chief,

PLOS One.

Dear Sir/Madam,

We thank you for your decision on the manuscript **PONE-D-24-59070R1** titled **“Molecular Detection and Antibiotic Resistance of Diarrheagenic *Escherichia coli* from Street food and Water in Mukuru slums, Nairobi County”.**

We also appreciate the reviewers’ constructive and insightful comments. We have carefully considered the comments and revised the manuscript accordingly. We hope that with these improvements, the revised manuscript will satisfy the journal’s requirements for publication. The following is a point-by-point list of our responses to the comments and changes made.

1. If the authors have adequately addressed your comments raised in a previous round of review and you feel that this manuscript is now acceptable for publication, you may indicate that here to bypass the “Comments to the Author” section, enter your conflict of interest statement in the “Confidential to Editor” section, and submit your "Accept" recommendation.

Reviewer #4: (No Response)

Reviewer #5: All comments have been addressed

**Response:** We thank the reviewers for their thoughts on the manuscript as it has greatly improved the manuscript.

2. Is the manuscript technically sound, and do the data support the conclusions?

The manuscript must describe a technically sound piece of scientific research with data that supports the conclusions. Experiments must have been conducted rigorously, with appropriate controls, replication, and sample sizes. The conclusions must be drawn appropriately based on the data presented.

Reviewer #4: Partly

Reviewer #5: Yes

**Response:** We appreciate the comments from reviewers. We believe that the revised data in the manuscript supports the conclusion.

3. Has the statistical analysis been performed appropriately and rigorously?

Reviewer #4: No

Reviewer #5: Yes

**Response**: We wish to thank the reviewers again for taking time to review the manuscript. We believe that the statistical analysis done has provided in the manuscript and is rigorous.

4. Have the authors made all data underlying the findings in their manuscript fully available?

The PLOS Data policy requires authors to make all data underlying the findings described in their manuscript fully available without restriction, with rare exception (please refer to the Data Availability Statement in the manuscript PDF file). The data should be provided as part of the manuscript or its supporting information, or deposited to a public repository. For example, in addition to summary statistics, the data points behind means, medians and variance measures should be available. If there are restrictions on publicly sharing data—e.g. participant privacy or use of data from a third party—those must be specified.

Reviewer #4: Yes

Reviewer #5: Yes

**Response:** We wish to thank the reviewers again for taking time to review the manuscript. We believe that the statistical analysis done has provided in the manuscript and is rigorous

5. Is the manuscript presented in an intelligible fashion and written in standard English?

PLOS ONE does not copyedit accepted manuscripts, so the language in submitted articles must be clear, correct, and unambiguous. Any typographical or grammatical errors should be corrected at revision, so please note any specific errors here.

Reviewer #4: Yes

Reviewer #5: Yes

**Response:** We appreciate the comments from reviewers.

6. Review Comments to the Author

Please use the space provided to explain your answers to the questions above. You may also include additional comments for the author, including concerns about dual publication, research ethics, or publication ethics. (Please upload your review as an attachment if it exceeds 20,000 characters

Reviewer #4: In this study, the authors investigated various characteristics of Escherichia coli isolated from food and street water, but the results were not statistically analyzed properly. Comments for improving the manuscript are included within the main text.

1.	Statistical analysis should be included in the abstract .

**Response:** We appreciate the reviewer for this comment and agree with the suggestions. We have revised the abstract and included the statistical analysis in line 20-22. “We have included the statistical methods used in the revised manuscript.

2. What were the positive controls.

**Response:** We appreciate the reviewer for this comment .We have revised the manuscript and included the positive controls in line 123.“

3.Was the difference in the prevalence of *E. coli* among the samples significant.

**Response:** We appreciate the reviewer for this comment . A Pearson’s chi-square test confirmed a statistically significant association between the type of sample and *E. coli* positivity (χ²(4) = 79.71, p < 0.001). This has been included in line 174-175.

4. Present and electrophoresis gel image

**Response:** We appreciate the reviewer for this comment. A gel image has been included as a supporting document.

5. Was the difference in the pathotypes statistically.

**Response:** We appreciate the reviewer for this comment There was a statistical significant association between *E. coli* pathotype and detection rate, as determined by Pearson’s chi-square test (χ²(4) = 63.11, p < 0.001. This has been included in line 181-182.

6. Present and electrophoresis gel image

**Response:** We appreciate the reviewer for this comment. A gel image has been included as a supporting document.

**Reviewer #5:**

The manuscript presents a cross-sectional study assessing the occurrence, pathotyping, and antimicrobial resistance (AMR) profiles of diarrheagenic Escherichia coli (DEC) in street food and water samples collected from the Mukuru informal settlements in Nairobi, Kenya. The topic is timely and important, especially considering the growing public health threat posed by antimicrobial resistance and fecal contamination in low-resource urban settings.

Although I was not involved in the initial round of peer review for this manuscript, it appears that the authors have adequately addressed the concerns and suggestions raised during the initial review process. The revisions are thoughtful, and the manuscript has been significantly improved as a result. However, I’ve the following suggestions:

1. The figure captions and corresponding explanations are not adequate. Provide specific details corresponding to each figure.

**Response:** We appreciate the reviewer for this comment. We have included captions and corresponding explanations to each figure.

2. Provide appropriate Axis titles for figures 2,3,4,6 and 7.

**Response:** We appreciate the reviewer for this comment. We have provided appropriate axis titles for each figure.

3. Streamline content in the Results and Discussion section to avoid redundancy and focus on key insights specific to this setting.

**Response:** We appreciate the reviewer for this comment. We have streamlined the content in the results and discussion section to avoid redundancy and focus on key insights specific to this setting.

Thank you for your consideration.

Kind regards,

Sheillah Mundalo
